# Sequential process optimization for a digital light processing system to minimize trial and error

**DOI:** 10.1038/s41598-022-17841-5

**Published:** 2022-08-08

**Authors:** Jae Won Choi, Gyeong-Ji Kim, Sukjoon Hong, Jeung Hee An, Baek-Jin Kim, Cheol Woo Ha

**Affiliations:** 1 Advanced Joining and Additive Manufacturing R&D Department, Korea Institute of Industrial Technology, 113-58, Seohaean-ro, Siheung-si, 15014 Republic of Korea; 2 Department of Mechanical Engineering, BK21 FOUR ERICA-ACE Center, Hanyang University, 55 Hanyangdaehak-ro, Ansan, 15588 Republic of Korea; 3 Department of Food and Nutrition, KC University, 47, 24-Gil, Kkachisan-ro, Seoul, 07661 Republic of Korea; 4 Green Chemistry and Materials Group, Korea Institute of Industrial Technology, Daejeon, Chungcheongnam-do 31056 Republic of Korea; 5 Department of Green Process and System Engineering, Korea University of Science and Technology (UST), Daejeon, Chungcheongnam-do 31056 Republic of Korea

**Keywords:** Mechanical engineering, Biomaterials, Techniques and instrumentation

## Abstract

In additive manufacturing, logical and efficient workflow optimization enables successful production and reduces cost and time. These attempts are essential for preventing fabrication problems from various causes. However, quantitative analysis and integrated management studies of fabrication issues using a digital light processing (DLP) system are insufficient. Therefore, an efficient optimization method is required to apply several materials and extend the application of the DLP system. This study proposes a sequential process optimization (SPO) to manage the initial adhesion, recoating, and exposure energy. The photopolymerization characteristics and viscosity of the photocurable resin were quantitatively analyzed through process conditions such as build plate speed, layer thickness, and exposure time. The ability of the proposed SPO was confirmed by fabricating an evaluation model using a biocompatible resin. Furthermore, the biocompatibility of the developed resin was verified through experiments. The existing DLP process requires several trials and errors in process optimization. Therefore, the fabrication results are different depending on the operator’s know-how. The use of the proposed SPO enables a systematic approach for optimizing the process conditions of a DLP system. As a result, the DLP system is expected to be more utilized.

## Introduction

Vat photopolymerization (VPP) is an additive manufacturing technology that can be used in various fields such as medicine, jewelry, and industry^1–4^. As it can be applied from the nanoscale to macroscale, the continuous demand and related research have increased^5–9^. Digital light processing (DLP), a VPP method, is an additive technology that uses a light source to photopolymerize a single layer of a three-dimensional (3D) structure by single exposure. The production speed is fast because it uses the digital mirror device (DMD) module of the beam projector to produce a pattern on a flat surface^10^. The DLP system uses a liquid photocurable resin, which is a synthetic organic material primarily composed of monomers, oligomers, photoinitiators, and additives. The photoinitiator receives the specific wavelength energy and is activated to generate a free radical to form a polymer bond with the monomer and oligomer^11–13^.

The liquid photocurable resins used may have different process conditions depending on the manufacturer, even if they are based on the same polymer; therefore, the identification of optimized process conditions for each resin is vital. The understanding of the material properties and curing conditions of newly developed resins is necessary to apply them to the DLP system. However, for many commercially available and developed resins, data are only provided on the curing thickness according to the exposure time of a specific wavelength. Therefore, unnecessary trials and errors are required for successful production. A logical and efficient workflow should be followed to reduce waste of resins and time and obtain high-quality products.

The VPP method is divided into bottom-up and top-down approaches according to the build plate and vat configuration, as shown in Fig. S1^14^. In the bottom-up approach, the build plate rises from the bottom of the vat to perform the process, as shown in Fig. S1a. The bottom-up method is primarily used for projection types, such as DLP systems^15,16^. In the top-down approach, the process proceeds as the build plate descends from the top of the vat to the bottom, as shown in Fig. S1b. The top-down method is widely used in laser scanning types, such as stereolithography apparatus^17,18^.

**Figure 1:**
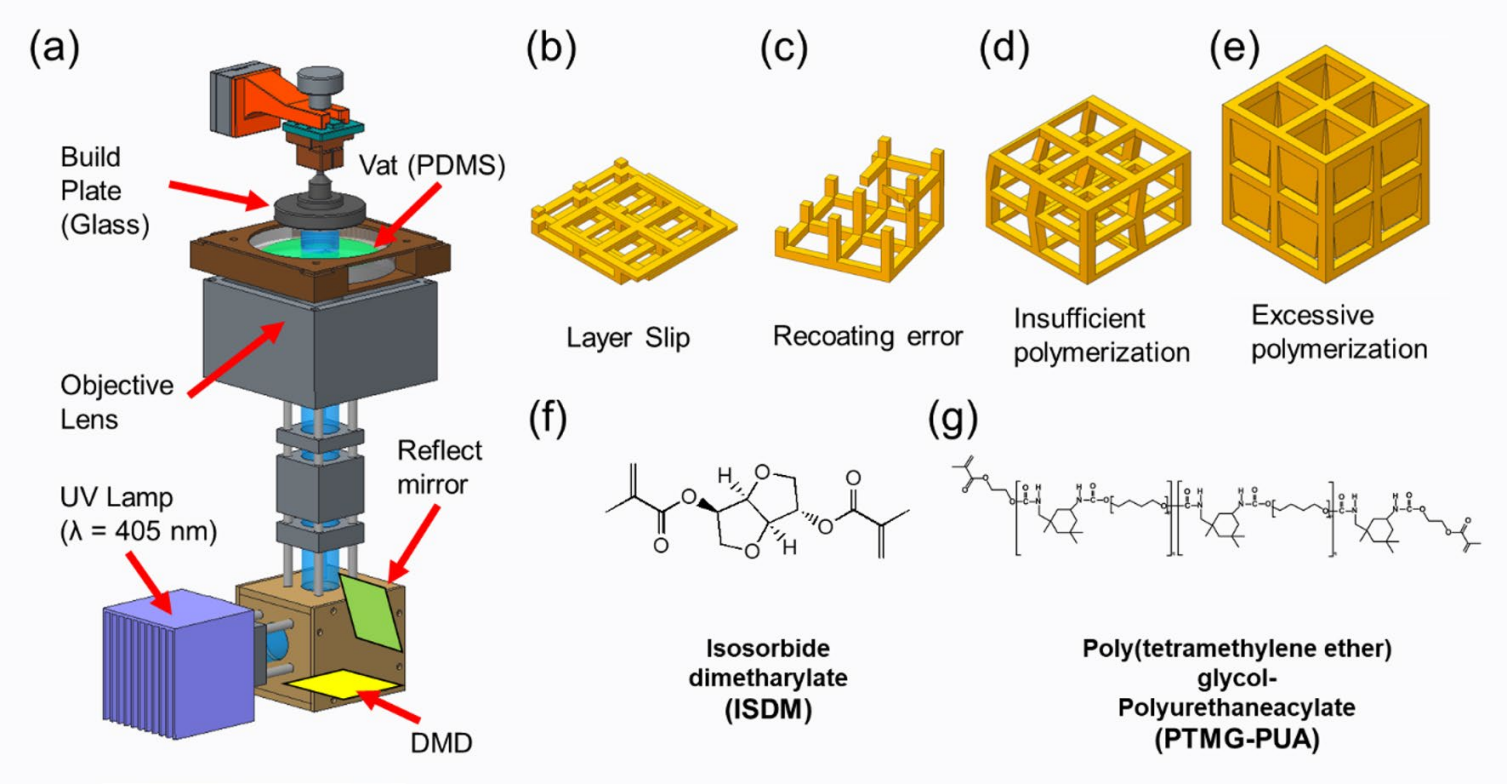
(**a**) Schematic diagram of DLP system. (**b**) Layer slip error due to poor build plate adhesion of the first layer. (**c**) Incomplete fabrication due to recoating error. (**d**) Shape distortion of the structure due to insufficient polymerization. (**e**) Over-curing due to excessive polymerization. (**f**) Chemical structure of isosorbide dimethacrylate (ISDM), the primary material of photocurable resin. (**g**) Chemical structure of poly(tetrameghylene ether) glycol-polyurethane acrylate (PTMG-PUA) as an additive material to improve the properties of phtocurable resins.

Most DLP systems use the bottom-up approach to fabricate components upside down, which has three advantages. First, the bottom-up approach enables a more precise control of the layer thickness than the top-down approach^19^, such that high-precision parts can be manufactured^20^. Second, the bottom-up method does not require a re-coater for the recoating mechanism because the resin is automatically recoated between fabrication processes by the build plate movement^21^. Third, the bottom-up approach is more cost-effective than the top-down approach. The top-down approach requires a large amount of resin because the fabrication area is immersed in resin^22^. In the top-down method, photopolymerization is selectively performed on the top surface of the vat, as shown in Fig. S1b. The 3D structure is fabricated as the build plate descends toward the bottom of the vat. Therefore, the resin must be filled to the top surface of the vat, to where the focus of the light source is set. The height of the vat becomes the maximum height fabricated by the top-down approach, and the higher it is, the more resin is required. Conversely, the bottom-up approach selectively performs photopolymerization on the bottom surface of the vat, as shown in Fig. S1a. If the resin covers the fabrication area, the bottom-up method can be used with a small amount of resin. In addition, as the maximum build height is determined by the rise height of the build plate, there are low restrictions on the fabrication height. Therefore, the bottom-up method enables fabrication with a small amount of resin, and cleaning is easy^23,24^. Table S1 shows a comparison of the top-down and bottom-up approaches. However, some common problems can occur with these DLP systems based on the bottom-up approach, including poor initial adhesion, poor recovery of resins, and over-curing of overhang structures, as depicted in Fig. 1b–e. These three problems are examined in more detail as follows.

First, an initial fabrication failure problem may occur if the adhesion between the build plate and the first layer is weak. The DLP process proceeds with repeated up-and-down movements of the build plate. The build plate moves down for the curing of the layer and up to prepare for the curing of the next layer. In this process, the first layer is continuously affected by tensile force. Therefore, if the first layer has weak adhesion to the build plate, the 3D structure may detach during the DLP process, or layer alignment may be a problem owing to layer sliding, as shown in Fig. S2a. Conversely, additive manufacturing is possible if the adhesion between the build plate and the first layer is strong. However, an excessively strong adhesion of the first layer causes strong cross-linking of the polymer. This leads to shrinkage and deformation of the cured layer, as shown in Fig. S2b.

Second, poor recoating of the photocurable resin can occur. Resin recoating is required for additive manufacturing in bottom-up DLP systems before ultraviolet (UV) exposure. The resins on the vat are automatically arranged and coated when the z-stage is elevated using the bottom-up approach. The viscosity of the resin influences the recoating result at this stage. When using a DLP system, the viscosity of the photocurable resin is an important property that affects the production speed and shape distortion. In the recoating process for curing the next layer, the high-viscosity photocurable resin prevents smooth recoating and increases the time required for fabrication.

Third, in a DLP system, over-curing occurs when a light of excessive energy penetrates the resin to a depth greater than the thickness of the layer, as shown in Fig. S2e. The more transparent the resin, the greater the over-curing. As a result, the inside of the undercut structure, such as the microchannel, becomes blocked^25^. There are several methods to change the layer thickness and solve over-curing: UV intensity, exposure wavelength, exposure time, and resin^26–28^. The most convenient are to change the UV intensity and exposure time. However, the reduction of these parameters to improve over-curing causes low interlayer adhesion, and the strength of the product is lowered, or shape implementation is complicated. These three problems should be addressed for the stable fabrication of an open channel or undercut structure.

In most studies, only the curing thickness according to the exposure energy of the photocurable resin was verified, and manufacturing has been attempted^29,30^. However, it is not easy to achieve successful production under these conditions. Furthermore, although many studies suggest a solution by focusing only on the specific problem^23,25,31^, such as cure depth, studies on the integrated management of the three representative problems are scarce. Logical and efficient optimization can reduce the possibility of failure, increase the yield of the process, and reduce processing time and cost. The existing DLP system often depends on the experience and expertise of skilled workers, and requires several trials and errors in process optimization.

Therefore, a sequential process optimization (SPO) for application to a DLP system is proposed in this study. The SPO was systematized to minimize trial and error, considering the three problems previously described. A 3D microstructure was fabricated using the process conditions derived from this, and the biocompatibility of the developed resin was confirmed through cell culture. A systematic approach for optimizing the process conditions of DLP printing was possible when using the SPO proposed in this study. Thus, the application range of the DLP system was increased by reducing the risk of process failure and waste of resources and time.

## Methods

### DLP system

Figure 1a illustrates the schematics of the developed DLP system^32^. The DLP system used a 6.6 W optical powered 405-nm near-UV LED (CBT39, Luminus), full-HD (1920 × 1080) DMD module (DLPLCR6500EVM, Texas Instruments), and a 100-mm focal length infinity-corrected objective lens (2 × EO M Plan Apo, Edmund Optics). The optical system used an enhanced aluminum-coated reflection mirror (#63-171, Edmund Optics) and a 100-mm focal length achromatic doublet lens (AC254–100, Thorlabs) to handle the optical path and improve imaging such as spherical aberration. The width of a single mirror of a DMD module is 7.56 μm. The field of view for the developed DLP system is 14.52 × 8.16 mm^2^. A five-phase stepper motorized Z-stage (ZCVL630, Misumi) with a 1-μm resolution and 30-mm stroke was used for precision additive manufacturing. An auto-leveling build plate with a ball-head joint was used. The glass build plate reduced the reflection and scattering of light from the UV lamp. The DLP system developed used the bottom-up method, as it is advantageous for producing microstructures, uses a small amount of resin, and is easy to clean.

In the bottom-up method, the polymerized resin may adhere to the vat surface owing to the excessive separation force. This problem can be solved by coating the vat with polydimethylsiloxane (PDMS), as depicted in Fig. 1a, because the porous structure of PDMS creates an oxygen inhibition layer that supplies oxygen between the PDMS window and the interface of the resin to inhibit radical polymerization. Therefore, the vat was coated with PDMS to reduce the release force between the cured resin and the vat^5^.

### Materials

#### Synthesis of ISDM

All chemicals used in this study were as follows: isosorbide (1,4:3,6-dianhydro-D-sorbitol, 98%), methacrylate anhydride (94%), ethyl 4-N,N-dimethylaminobenzoate (EDMAB), tetrahydrofuran (THF, anhydrous, 99.9%), and magnesium sulfate (MgSO4, 99.5%) were purchased from Sigma-Aldrich. Triethylamine (TEA) and 4,4-dimethylaminopyridine (DMAP) were purchased from Daejung for isosobide dimethacrylate (ISDM). Poly(tetramethylene ether)glycol (PTMG, average Mn 1000 g/mol), isophorone diisocyanate (IPDI, 98%), and diphenyl (2,4,6-trimethylbenzoyl)phosphine oxide (TPO, Shinyoun Rad Chem Ltd.) were also purchased from Aldrich, Korea, for the synthesis of PTMG-based urethane acrylate. ISDM (Fig. 1f) was synthesized as shown in Fig. S4^33^.

#### Synthesis of PTMG-based PUA

**Figure 2:**
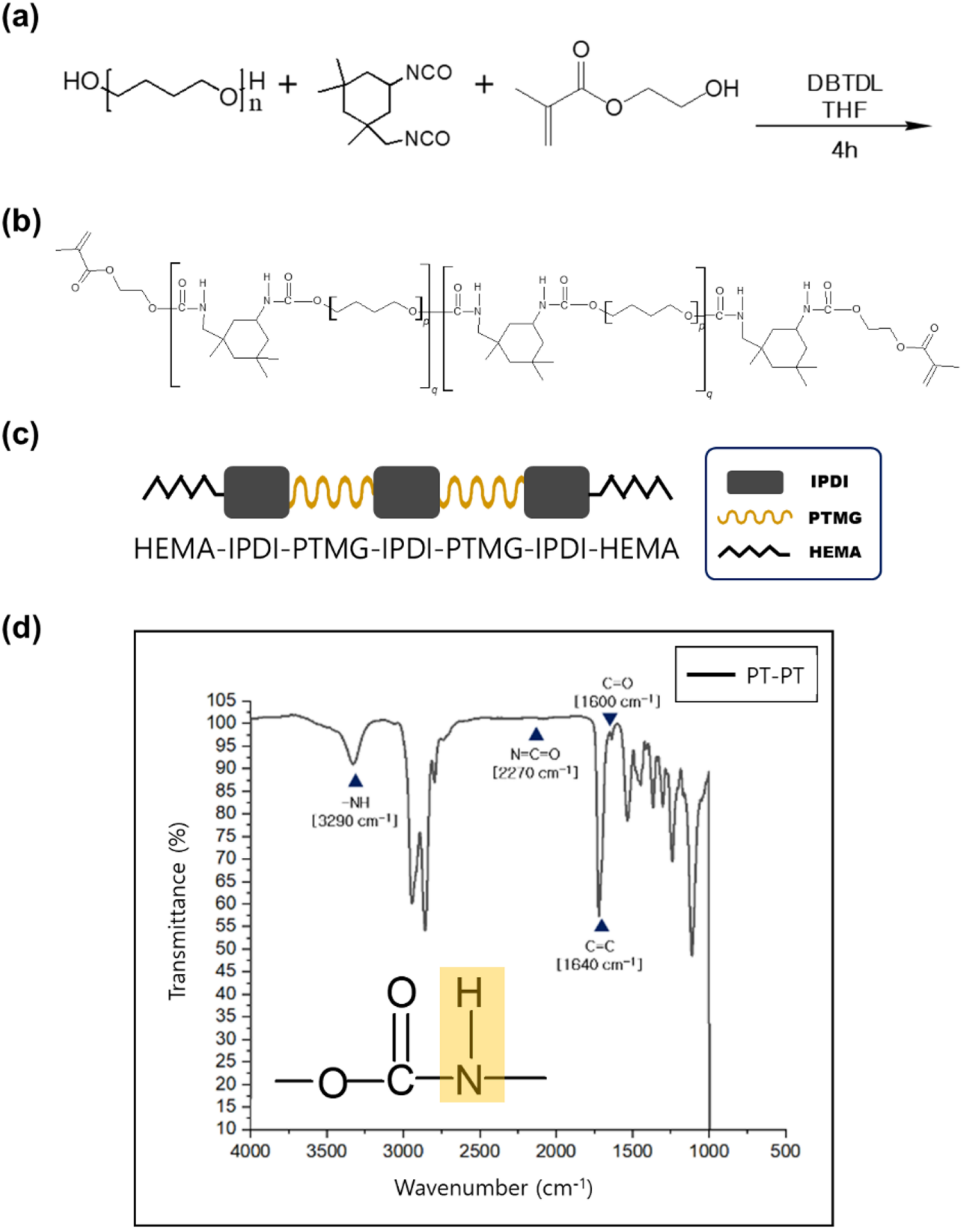
(**a**) Reaction scheme for PTMG-based PUA. (**b**) Square and spring symbols represent IPDI and PTMG (or HEMA), respectively, (**c**) PT–PT PUA structure of the final step and (**d**) FT-IR spectra of PTMG PUA.

The entire reaction proceeded in four steps by adding the reactants sequentially. In the first step, as shown in Fig. 2a, IPDI (14 mmol, 1 equivalents) and 0.01 g of DBTDL were placed in a three-necked flask under a nitrogen atmosphere and reacted at 60 °C for 10 min. A Fourier transform infrared (FT-IR) spectrometer was employed to confirm the functional groups of the target urethane acrylate. FT-IR spectroscopy was performed using a Thermo Scientific Nicolet™ 6700 spectrometer in the attenuated total reflection infrared mode. The analysis was conducted within the frequency range of 4000–400 cm^−1^ by co-adding 32 scans at a resolution of 2 cm^−1^.

PTMG (28 mmol, 2 equivalent) was added to IPDI with a tin catalyst and continuously stirred at 60 °C until the NCO functional peak (2270 cm^−1^) from FT-IR disappeared to obtain the PTMG-IPDI-PTMG structure with C=C (1640 cm^−1^) and C=O (1600 cm^−1^) functional groups in methacrylate. In step 2, as shown in Fig. 2b, IPDI (28 mmol, 2 equivalents) was added again to the PTMG-IPDI-PTMG structure and continuously stirred under nitrogen at 60 °C for 10 min until the OH peak (3360 cm^−1^) from FT-IR disappeared to obtain the IPDI-PTMG-IPDI-PTMG-IPDI structure. In step 3, 3.8262 g (29.4 mmol, 2.1 equivalents) of HEMA was added to the result compound in step 2, as shown in Fig. 2c. The final reaction proceeded at 40 °C until the NCO functional peak (2270 cm^−1^) disappeared from FT-IR, as shown in Fig. 2d, to obtain the target PTMG PUA with the HEMA-IPDI-PTMG-IPDI-PTMG-IPDI-HEMA structure.

#### Blend of ISDM and PTMG-based PUA

Isosorbide obtained by hydrogenation of glucose followed by dehydration of sorbitol has attracted considerable attention because of its rigidity and transparency^34–36^ as an alternative to bisphenol-A, an environmental hormone that is harmful to the human body^37^. Isosorbide is a renewable and non-toxic substance with good optical properties. However, owing to the brittleness of ISDM, there is a limit to additive manufacturing; thus, it was used in combination with other materials because it is colorless and transparent. PTMG is a material with excellent strength and abrasion resistance^38^. PUA has a high abrasion resistance and optical properties^39,40^. Thus, a blend of PTMG PUA and ISDM, as shown in Fig. 1f,g, may compensate for the limitation of isosorbide and enhance flexibility, which is the advantage of PTMG PUA. Accordingly, the effect of the weight ratio shown in Table S2 (0:10/2:8/8:2/10:0, w/w) between ISDM and PTMG PUA on tensile strength, elongation, light transmittance, and curing speed was investigated. As shown in Fig. S5, tensile strength and elongation were measured according to the ratio of ISDM and PUA (conditions 1, 2, 3 in Table S2). As shown in Figs. S6 and S7, light transmittance and curing speed were measured according to the ratio of ISDM and PUA (conditions, 2, 3, 4 in Table S2).

### Sequential process optimization

**Figure 3:**
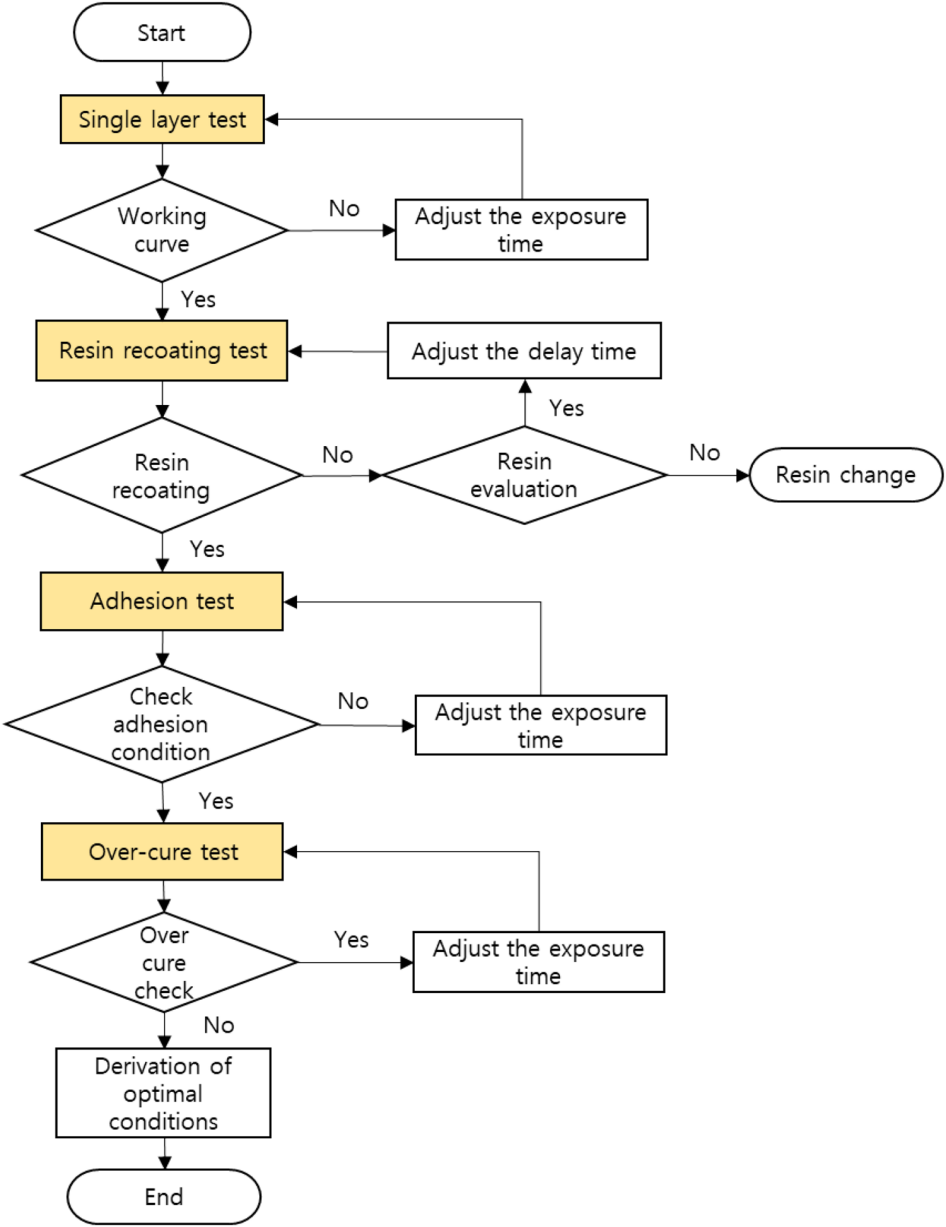
SPO workflow: single layer test, resin recoating test, adhesion test, over-cure test.

The SPO proposed in this study follows the workflow shown in Fig. 3. The first sequence of SPO is a single-layer test. The first sequence secures a dataset on the curing thickness of the resin according to the exposure energy, which is the most basic in the VPP method. During the process, the standard exposure energy required for the process is identified. The curing thickness of the resin according to the exposure energy has a significant impact on the fabrication of complex 3D microstructures. An undesired structure may be created if the cured thickness is too large or smaller than the layer thickness.

The second sequence is the resin-recoating test. In micro-additive manufacturing, careful consideration should be given to the use of recoaters that cause microstructure breakage^41^. Therefore, natural automatic recoating of the resin is essential in a bottom-up DLP system that does not require a recoater. However, due to this, air bubbles generated in the processing area remain inside the final product and may deteriorate the physical properties or cause shape errors outside the product. Therefore, the appropriate resin viscosity and process speed should be determined to prevent air bubbles and incomplete resin filling during the recoating process.

In the third sequence, i.e., the first layer adhesion test, the adhesion condition of the first layer is confirmed. This section discusses the adhesion between the build plate and the first layer. If the adhesion between the build plate and the first layer is too weak, delamination problems may occur during the initial fabrication. Conversely, if adhesion is too strong, fabrication is possible, but the structure may crack or deform with time. In this study, the layer adhesion test is conducted according to the exposure time to confirm the optimal adhesion. Optimal adhesion is checked simply by fabricating a layered pattern over exposure time and raising the build plate to process speed.

In the final sequence, i.e., the over-cure test, the appropriate overlap between the layers must be confirmed to minimize over-curing. An adequate overlap between layers is required for balanced fabrication^31^. If the overlap level is too low, the durability of the structure decreases. Conversely, if the overlap level is excessive, an unintended shape is produced owing to the over-cure. This is one of the reasons why it is challenging to fabricate overhang structures using a DLP system. Photocurable resins are made nontransparent by adding light absorbers or dyes to prevent over-curing^42^. Because the resin is nontransparent, the light penetration depth is limited, reducing the probability of over-curing. However, the use of additives causes changes in the physical properties of the raw material and deterioration of transparency. Consequently, it is challenging to observe internal structures, such as microchannels, and optical applications are limited. In this study, to maintain the properties of the raw material, the optimal overlap condition was determined using the exposure energy without adding other additives.

## Results and discussion

### Single-layer test

**Figure 4:**
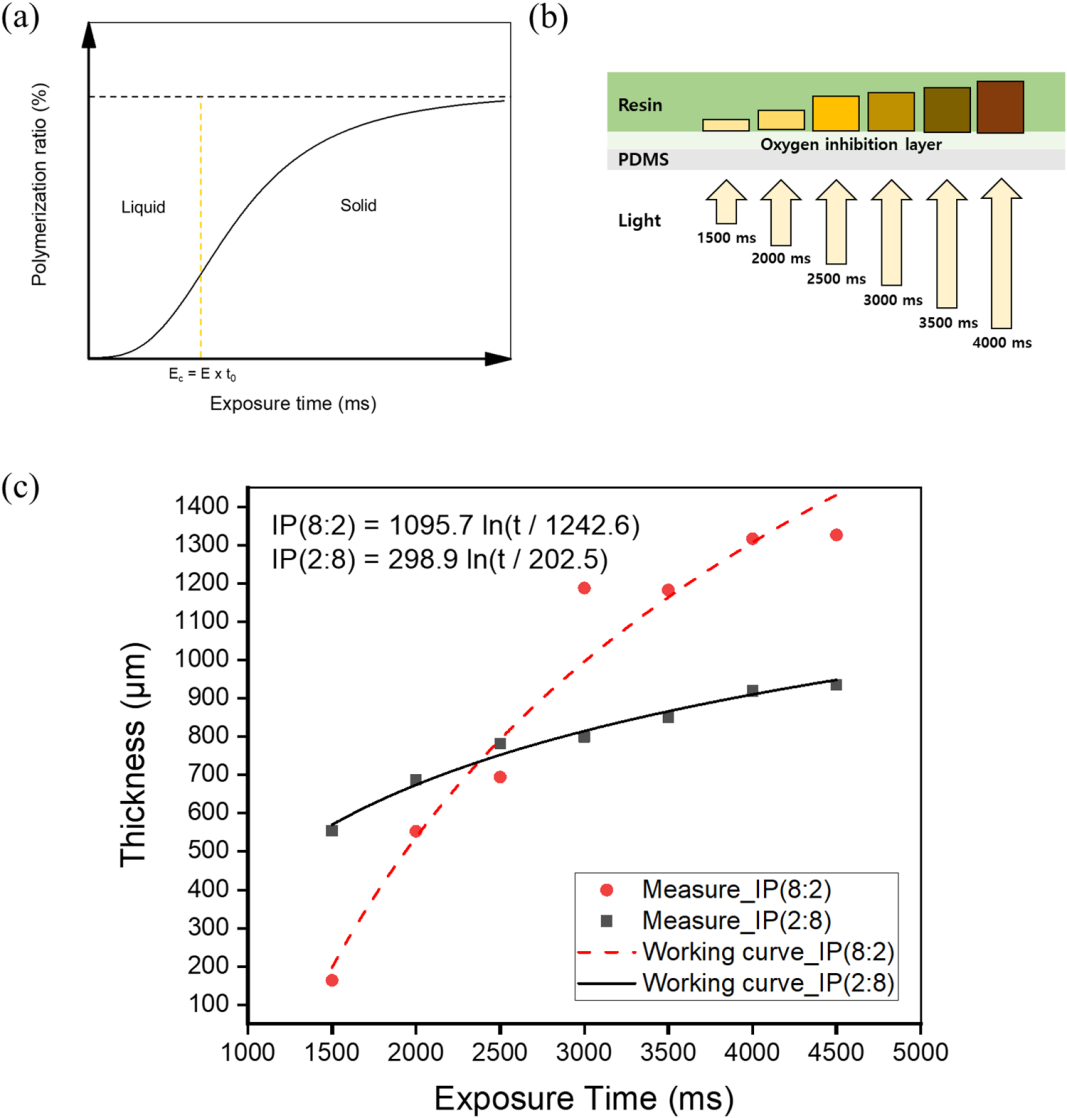
(**a**) Photopolymerization ratio and critical exposure energy according to the exposure time. (**b**) Single-layer photopolymerization according to the exposure time. (**c**) Single-layer thickness measurement result for each resin.

The cured thickness of the resin due to the photopolymerization reaction can be calculated and predicted mathematically. When UV light hits the resin, some light is scattered or absorbed at the resin surface. According to the Beer–Lambert law, the intensity of light decreases exponentially. Equation (1) is the working curve equation, which is derived by arranging the exposure equation obtained through the Beer–Lambert law in terms of energy, where *C*_*d*_ is the maximum curing thickness, *D*_*p*_ is the penetration depth of light, *E*_*max*_ is the maximum exposure energy of the resin surface, and *E*_*c*_ is the critical energy at which the resin phase changes into a solid. As shown in Fig. 4a, the critical energy *E*_*c*_ is the minimum energy required to initiate photopolymerization. According to Eq. (1), the curing properties of resins depend on two parameters: exposure energy and penetration depth^43,44^. As the amount of energy input and penetration depth increase, the cured thickness of the resin increases.

1 $${C}_{d}={D}_{p}\,\mathrm{ln}\left[\frac{{E}_{max}}{{E}_{c}}\right]$$

2 $${C}_{d}={D}_{p}\,\mathrm{ln}\left[\frac{E\cdot t}{E\cdot {t}_{0}}\right] = {D}_{p}\,\mathrm{ln}\left[\frac{t}{{t}_{0}}\right]$$

Based on Eq. (2), the curing thickness of the resin can be predicted using the exposure time as a process variable, where *t* is the exposure time, *t*_*0*_ is the critical time. As shown in Fig. 4b, the build plate was removed, and two types of newly developed resins were placed on the PDMS-coated vat. A square pattern of 2 × 2 mm^2^ was exposed to each resin with a light source with a wavelength of 405 nm from 1500 to 4500 ms at intervals of 500 ms. According to Eq. (1), the depth of penetrated UV light is proportional to the exposure time, because the maximum exposure energy depends on the time integral of the irradiance. To evaluate the thickness of the cured single layer according to various exposure times of UV light, the cured single layer was separated from the vat, washed with isopropyl alcohol (IPA), and the cured thickness was measured using an optical microscope (MS-12Z-L1215).

The curing thickness according to the exposure time of the two types of resins is shown in Fig. 4c. The curing thickness is controlled by adding a specific dye, photoinitiator, or UV absorber to the photocurable resin. However, in this case, lowering the transparency of the raw material is disadvantageous. Thus, the exposure energy was used to maintain the transparency of the photocurable resin.

The critical energy *E*_*c*_ is obtained by multiplying the critical time *t*_*0*_ and the illuminance *E*^41^. *E*_*c*_ is difficult to observe experimentally. This is because, in theory, the curing thickness is zero at *E*_*c*_. Therefore, prediction is a standard method for deriving *E*_*c*_^45^. A tendency line was obtained from the measured curing thickness, and the corresponding *x*-intercept value was assumed to be *t*_*0*_. The average light penetration depth *D*_*p*_ was obtained by substituting the measured *C*_*d*_, *t*_*0*_, and t values into Eq. (2). Consequently, the working curve equation of the resin was derived, as shown in Fig. 4c. Additional experiments were performed to verify the derived working curve equation, as shown in Fig. S8. The cured thickness is highly sensitive to exposure time with a large amount of ISDM. As shown in Fig. 4c, IP(2:8) had a considerably lower gradient than IP(8:2). This is because the photopolymerization levels of the ISDM and PTMG PUA differ according to the exposure energy. This result indicates that IP(8:2) enables a broader range of thickness control.

### Resin recoating test

**Figure 5:**
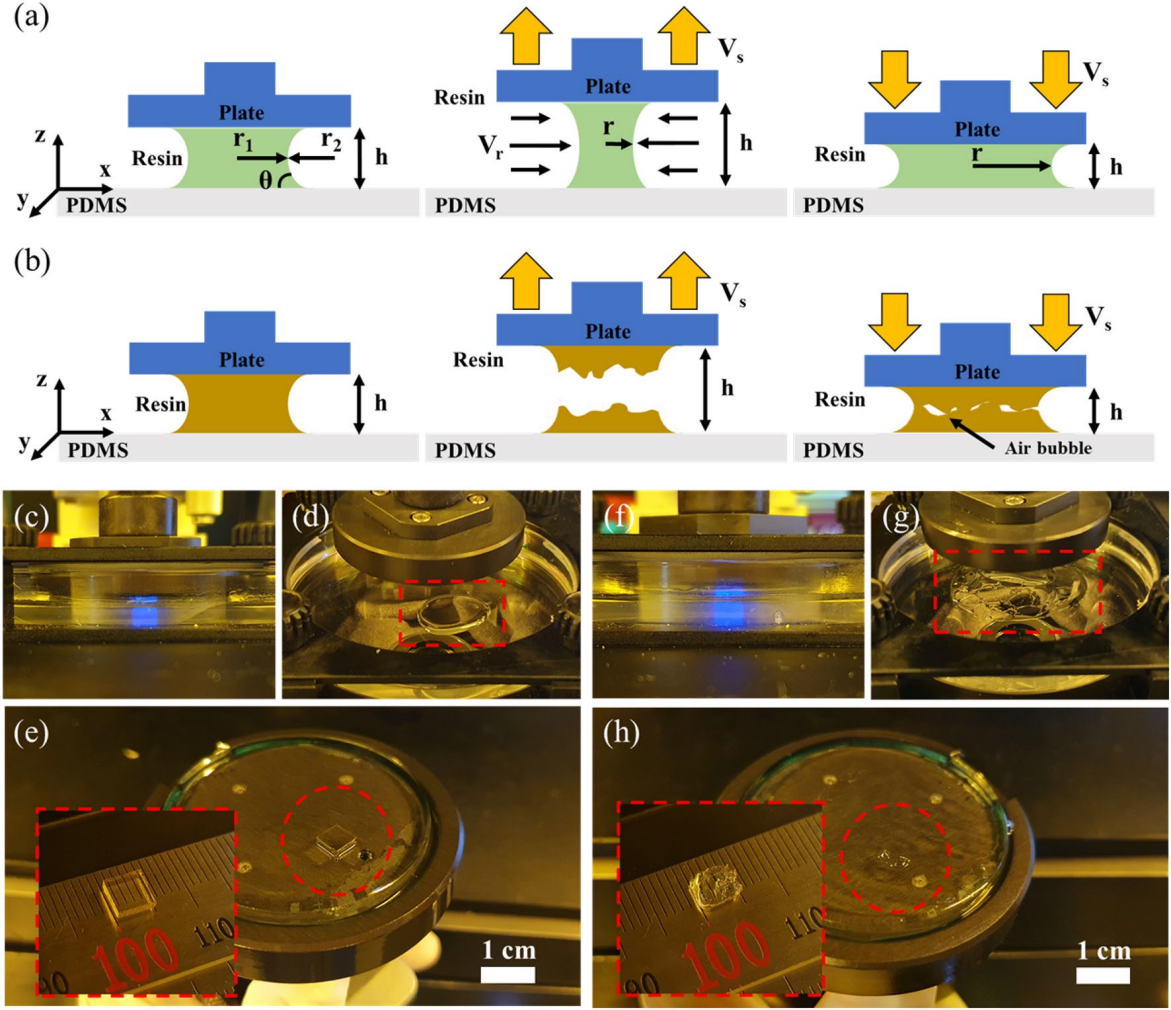
Behavior of (**a**) low-viscosity resin and (**b**) high-viscosity resin according to the movement of the build plate in the *z*-axis direction. (**c**,**d**) Photopolymerization and non-breaking resin interface of low-viscosity resin. (**e**) Samples without internal bubbles and shape errors due to stable recoating. (**f** **g**) Photopolymerization and breaking resin interface of high-viscosity resin. (**h**) Sample shape error due to poor recoating.

After one layer is fabricated in the DLP system, the build plate rises farther than the layer thickness in the *z*-axis direction, as shown in Fig. 5a. After the z-axis has stopped and a set delay time had passed, the build plate was lowered to the next layer position. In this process, resin settling behavior occurs. As such, the success of recoating is determined by the photocurable resin viscosity and the build plate speed.

In a DLP system, the viscosity of the photocurable resin is an essential parameter^46,47^. The higher the viscosity of the resin, the more time is required for recoating, and the possibility of air bubbles in the fabrication area increases, as depicted in Fig. 5b.

The vat coated with PDMS was located above the optical system, and a glass build plate was mounted on the *z*-axis stage and positioned parallel to the vat (Fig. 1a). Assuming that the vat and the build plate are parallel, the photocurable resin flows between infinitely large plates with very narrow gaps. Therefore, the resin flow between the vat and build plate can be assumed to be a fully developed laminar flow without time disturbance^48^. The capillary force dominates the force acting on the two plates and the resin because the effect of gravity is negligible for liquids between the micro/nanogap. A large negative pressure is generated at the liquid interface between the two plates at micro/nano spacing, which functions as a large adhesion force between the two plates^49,50^.

The adhesion between the build plate and vat is a critical factor to consider for successful fabrication in a bottom-up DLP system. The initial condition setting considering the adhesive force was managed in the following sequence. The current sequence conformed to the recoating condition according to the behavior of the resin between the two plates.

The recoating process proceeded in the DLP system, as shown in Fig. 5a,b. Figure 5a illustrates the use of a resin with an appropriate viscosity, and Fig. 5b illustrates the use of a high-viscosity resin. The internal pressure of the resin between the vat and build plate is expressed by Eq. (3):

3 $$\Delta p=T\left(\frac{1}{{r}_{1}}-\frac{1}{{r}_{2}}\right)$$

where *T* is the surface tension, *r*_*1*_ is the radius of the middle point between the vat and the build plate inside the resin, and *r*_*2*_ is the radius of curvature outside the resin. When the pressure inside the droplet is lower than the outside pressure (Δp < 0), the capillary adhesion pulls the two surfaces above and below. The generated capillary adhesive force is expressed by Eq. (4).

4 $$F=\frac{2TV\mathrm{cos}\,\theta }{{h}^{2}}$$

where *V* is the volume of the resin, *θ* is the contact angle, and *h* is the distance between the vat and the build plate. Assuming that the amount of evaporation of the resin is negligible, the numerator can be treated as a constant. Therefore, adhesion is most affected by the gap between the vat and build plate, and adhesion F decreases as the build plate rises^51^.

Assuming that the surface roughness of the vat and build plate is uniform when the build plate rises, the interface retreats in the radial direction of the build plate to maintain the resin volume and contact angle on the surface^52,53^, as depicted in Fig. 5a. The resin is recoated because of this flow, but the fingering effect and bubbles occur due to the instability of the interface depending on the viscosity of the resin^54^.

In a low-viscosity resin, the interface stability of resin-vat and resin-build plates is high because the flow rate *V*_*r*_ of the resin is greater than or equal to the rate of increase of the build plate speed *V*_*s*_. Accordingly, the movement of the resin and the build plate has high synchronicity, as depicted in Fig. 5a. However, for a high-viscosity resin, because *V*_*r*_ is smaller than *V*_*s*_, there is a mismatch in the resin movement for the build plate movement, causing the resin to separate, as depicted in Fig. 5b. This causes bubbles in the resin and fabrication failure.

Owing to the fast fabrication speed of the DLP system, the photocurable resin should behave flexibly between the vat and build plate. The resin flow between the two plates is an incompressible laminar flow, and the velocity distribution *V*_*r*_ of the resin is expressed by Eq. (5)^55^:

5 $${v}_{r}=\frac{z\Delta p}{\mu L}\left(h-z\right)$$

where *z* is the vertical distance from the vat, *L* is the *x*-direction length of the curing area, and *μ* is the viscosity of the resin. The maximum velocity *V*_*max*_ occurs at point *h/2*, and is expressed by Eq. (6):

6 $${v}_{max}=\frac{\Delta p{h}^{2}}{4\mu L}$$

As previously mentioned, the flexibility of the resin flow during the rise and fall of the build plate is critical for effective recoating. Accordingly, a difference between the build plate speed *V*_*s*_ and the resin speed *V*_*max*_ closer to 0 is optimal. When the build plate movement time is *t*_*b*_, the speed *V*_*s*_ can be expressed as *h/t*_*b*_. If this is summarized for time *t*_*b*_ together with Eqs. (6), (7) is identical.

7 $${t}_{b}=\frac{4\mu L}{\Delta ph}$$

According to Eq. (7), the higher the viscosity of the resin, the more time is required for the build plate movement. Therefore, the use of a low-viscosity resin is advantageous for rapid manufacturing and stable recoating in a DLP system. The relationship between the build plate speed and viscosity also affects the build area, but it was not considered in this study because the maximum build area was as small as 13 × 7.5 mm^2^.

The manufacturability based on the difference in viscosity was confirmed through experiments using the low-viscosity IP(8:2) and high-viscosity IP(2:8) resins selected in this study. A 5 × 5 × 1 mm^3^ sample was fabricated with the DLP system, as shown in Fig. 5c,f under a layer thickness of 100 µm, z-lift distance of 1 mm, and z-lift speed of 100 mm/min. Because the purpose of this experiment was to confirm the effect of viscosity, the exposure time was set to 2500 ms such that the resin could be sufficiently photopolymerized. The time required for the one-layer process cycle under this condition was 5.9 s.

A distinct difference in resin behavior according to viscosity was confirmed when the build plate returned to its origin. The IP(8:2) resin maintained a mass on the vat without breaking the interface during the process, as shown in Fig. 5d. The sample in Fig. 5e was produced without internal bubbles or shape errors.

In contrast, in the IP(2:8) resin, as shown in Fig. 5g, the interface was broken on the vat and separated, causing air bubbles during the process, interfering with resin filling, and causing manufacturing defects, as shown in Fig. 5h. For high-viscosity IP(2:8) resin, the results can be improved if the z-lift speed is lowered and sufficient recoating time is considered. However, this requires a very long process time, which limits the fast manufacturing of the DLP system. Therefore, IP(2:8) resin was no longer considered in the subsequent sequence, and optimization was performed using IP(8:2) resin.

### First layer adhesion test

To reliably attach the first layer to the build plate, the adhesive force between the build plate and the first layer must be greater than the stress that occurs when the build plate is raised for the next layer. The force that occurs when two plates are separated between a viscous fluid is defined as the Stefan adhesion in Eq. (8)^56,57^:

8 $$F=\frac{3\pi \mu {R}^{4}}{2{h}^{3}}\frac{dh}{dt}$$

where *R* is the radius of the separating plates, h is the distance between the two plates, μ is the viscosity of the fluid, and *dh/dt* is the rate of separation. Equation (8) indicates that the force applied to the previous layer is linearly proportional to the separation speed as the build plate moves to the next layer. Previously, the relationship between the processing time and resin viscosity at a z-lift speed of 100 mm/min was confirmed through the resin-recoating test sequence. The z-lift speed of 100 mm/min was the same as that of Eq. (8).

**Figure 6:**
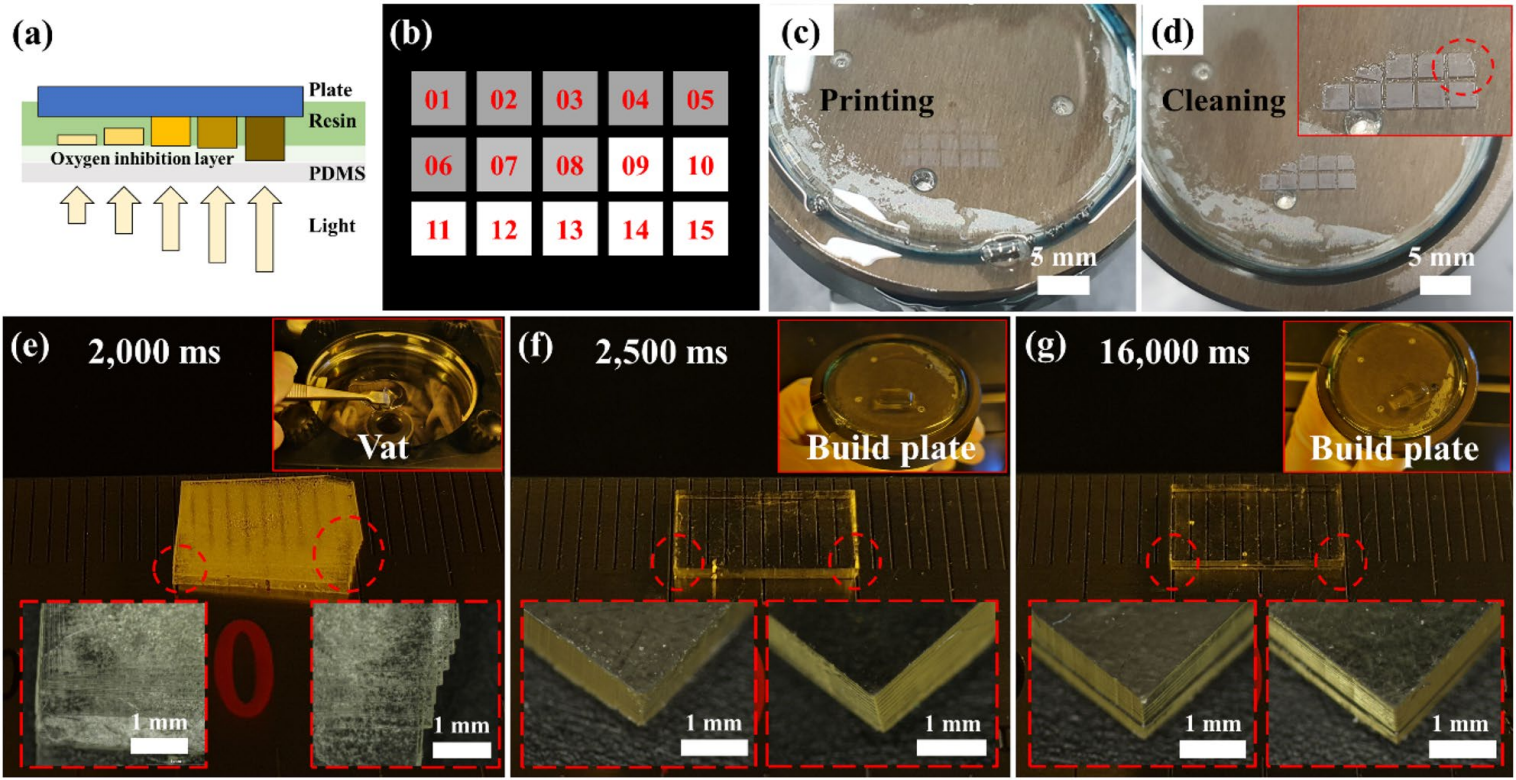
(**a**) Schematic diagram for the first layer adhesion test. (**b**) 3 × 5 square image pattern for adhesion test according to the exposure time. (**c**) First layer fabrication according to the exposure time. (**d**) After cleaning the build plate, the exposure time was confirmed to be suitable for first layer adhesion. (**e**) Failure to build plate adhesion due to insufficient exposure time. Misalignment of each layer is observed. (**f**) Sample fabricated by applying the optimal first layer exposure time. (**g**) First layer with excessive exposure time applied, causing dimensional accuracy failure and deformation due to step difference.

To identify the adhesion condition of the first layer at a separation speed of 100 mm/min, 2 ml of IP(8:2) resin was placed into the vat, as depicted in Fig. 6a, and the build plate was set at the starting position. Figure 6b illustrates that a 2 × 2 mm^2^ square image was generated as a 3 × 5 array. Each image was cured on the build plate by sequential exposure to 405 nm light from 1600 to 3000 ms at 100 ms intervals. After the process was completed, the build plate was raised at a separation speed of 100 mm/min. The cured pattern on the build plate shown in Fig. 6c was washed with IPA, and a distinct pattern was observed, as shown in Fig. 6d.

The pattern not attached to the build plate and that remaining on the build plate are confirmed in Fig. 6d. Patterns that do not adhere to the build plate are due to insufficient adhesion to resist the stresses caused by the separation speed. A stable adherence to the build plate was achieved from the tenth pattern, indicating that the first layer required 2500 ms or longer exposure time.

A 10 × 5 × 1 mm^3^ sample was prepared to confirm whether the first-layer exposure conditions were appropriate for the DLP system. Figure 6e illustrates the case of insufficient adhesion. The first layer applied the 2000 ms condition, and the remaining layers were fabricated with a layer height of 100 µm under the 1600 ms condition. In the working curve of the single-layer test, 2000 ms was a sufficient time for photopolymerization. However, the 2000 ms condition provided insufficient energy for build plate adhesion; thus, the process was terminated with the sample floating in the vat, as shown in the inset image of Fig. 6e. Samples were created as unaligned layers because when the initial layer adhesion failed, additional layer fabrication caused layer slip.

In Fig. 6f, 2500 ms derived through experimentation was applied as the optimal first-layer condition, and the remaining layers were fabricated with a layer height of 100 µm under the 1600 ms condition. The sample was fabricated in a state of adhesion to the build plate, as depicted in Fig. 6f. Each layer of the sample was aligned during fabrication. Finally, in Fig. 6g, the first layer condition was applied at 16,000 ms, ten times the process condition (recommended as the first layer conditions for commercial equipment).

The sample was adhered to a build plate during the fabrication. However, as shown in Fig. 6g, a step difference was observed between the first and subsequent layers, caused by over-curing owing to the excessive exposure time of the first layer. A step difference affects the dimensional accuracy and causes product deformation in the future. Therefore, confirming the first-layer adhesion condition using the method proposed in this sequence is helpful for successful fabrication. The optimized exposure time of the first layer adhesion for IP(8:2) was 2500 ms.

### Over-cure test

**Figure 7:**
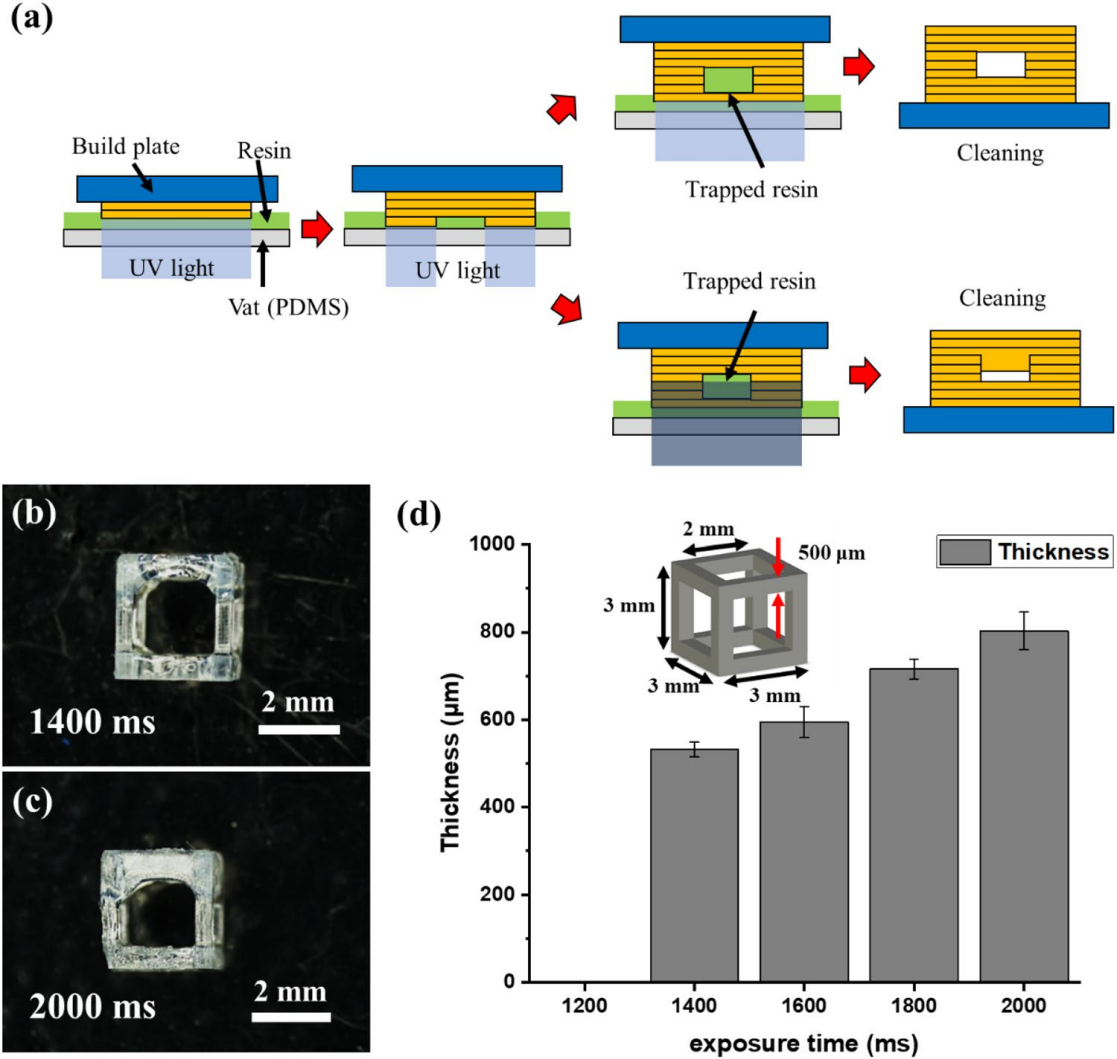
(**a**) Principle of over-cure in DLP system. (**b**) Lattice structure manufactured with 1400-ms exposure time. (**c**) Over-cured lattice structure manufactured with 2000-ms exposure time. (**d**) Measurement of over-cure of lattice structure overhang according to the exposure time.

Fabricating overhang structures using the VPP method remains challenging, particularly when the top angle is almost parallel to the build plate. As shown in Fig. 7a, the resin trapped in the structure causes an additional photopolymerization reaction due to UV transmission, causing an over-curing problem^58^. As the size of the structure decreases, the inside is clogged, and a sizeable dimensional error occurs.

IP(8:2) resin with a suitable viscosity for the DLP system was selected based on the resin recoating test. Furthermore, the first layer adhesion test performed in the third sequence confirmed that the initial exposure condition for stable attachment to the build plate was 2500 ms. Exposure time conditions were selected by referring to the working curve of the first sequence. The experiment was conducted with exposure time conditions set at 200-ms intervals from 1200 to 2000 ms. In the 2000-ms condition, up to five times the exposure energy can be produced compared with the laminate thickness.

A 3 × 3 × 3 mm^3^ lattice structure with a line width of 500 μm was fabricated under the conditions of a layer thickness of 100 μm, z-lift speed of 100 mm/min, and first-layer exposure time of 2500 ms. The fabricated lattice structure was washed with IPA and post-processed for 3 min in a UV oven with a wavelength of 405 nm. The thickness of the upper frame of the manufactured lattice structure was measured using an optical microscope (MS-12Z-L1215, SEIWA Optical), as shown in Fig. 7b,c.

The results for each condition are shown in Fig. 7d. Under the 1200-ms condition, as the exposure energy was insufficient to cause a photopolymerization reaction, the fabrication was unsuccessful. In contrast, at the 2.000-ms condition, over-curing of up to 1.6 times the design thickness of 500 μm occurred owing to excessive exposure energy. Therefore, the optimal exposure time was 1400 ms, 5% of the design thickness was overcured, and this value was the optimal overlap condition.

However, under these process conditions, despite reducing over-curing, the strength and durability of the structure immediately after fabrication were low because the bonding force between each layer was weak. Thus, post-processing in a UV oven after IPA cleaning was essential to improve the low strength and durability. UV curing also induced a complete reaction of the photoinitiator remaining on the structural surface. Therefore, UV curing improved strength and durability and reduced the biological toxicity.

### Evaluation and application

**Figure 8:**
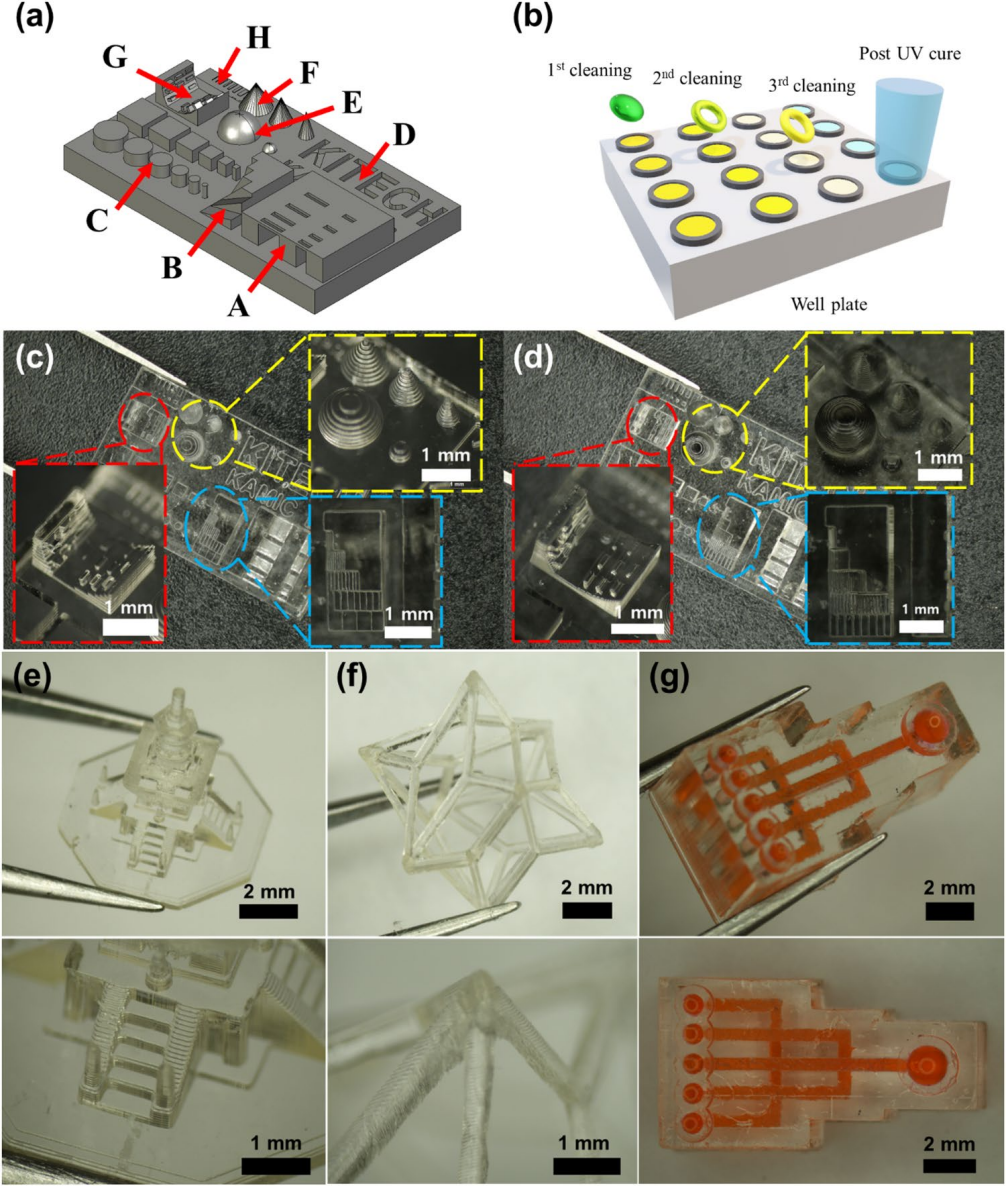
(**a**) Test artifact for verification of process conditions. (**b**) Conceptual diagram of three-step cleaning of DLP production sample. Test artifacts of (**c**) 100-μm layer and (**d**) 50-μm layer thickness conditions. (**e**) Dabo tower scaled down to 6/10,000. (**f**) Surface vertex centroid structure. (**g**) 3D microfluidic chip.

The optimal process conditions for the developed resin were obtained through SPO, as previously described. The test artifact for confirming manufacturability was designed to evaluate whether the obtained conditions were suitable, as depicted in Fig. 8a. The test artifact was divided into eight sections from A to H to confirm manufacturability. Section A confirmed the manufacturability of the overhang structure according to the *x*–*y* aspect ratio, and Section B evaluated the fabrication precision according to the angle. The production of cylinders and square pillars according to the *x*–*y* aspect ratio was verified in Section C. Section D confirmed the implementation of the engraved pattern according to the width, and section E evaluated the manufacturing precision of the spherical shape. Section F evaluated the fabrication of the cone shape according to the angle, and section G confirmed the manufacturability of the cantilever beam according to the angle.

In the VPP method, cleaning after manufacturing is also important and must be carefully performed because damage may occur in microstructures during cleaning. The removal of the uncured resin remaining on the structure safely and accurately without damage was performed through a three-step cleaning process, as depicted in Fig. 8b. A well plate or sample bottle was prepared to contain the fabricated structure and IPA for washing. The first wash removed most of the resin on the surface, the second wash was performed by transferring the structure to an uncontaminated IPA, and the same method was used in the third wash to remove the uncured resin inside the structure.

Although vibration using ultrasonic waves facilitates cleaning, excessive vibrations can cause damage to the structure and require care. The structures cleaned in the third step were cured using UV light. Because the microstructure before post-UV curing is not durable, it may be damaged when wiping the IPA on the surface. Therefore, post-UV curing was performed with the microstructure immersed in IPA or deionized (DI) water to complete the final curing process without damage or distortion, as depicted in Fig. 8b.

Figure 8c shows a test artifact manufactured using the process conditions derived from the proposed SPO. A test artifact manufactured with a layer thickness of 100 µm was produced without problems in most areas. However, a low resolution was observed in slanted or curved structures (e.g., sections B, E, F, and G) owing to the high layer thickness. The resolution was improved by generating a test artifact with a layer thickness of 50 μm. Process conditions with a layer thickness of 50 µm were inferred from the first and last sequences. Figure 8d illustrates the manufactured test artifact, where the resolution of the slanted or curved structure was improved. The process conditions obtained by the SPO were suitable for fabricating a complex 3D structure, as depicted in Fig. 8e–g.

### Biocompatibility test

Additional experiments were conducted using a commercial photocurable resin to confirm whether the proposed SPO is universally applicable. Spot-HT-blue (Spot-A Materials, Barcelona, Spain) and OrmoComp (Micro-resist Technology GmbH, Germany) were used as commercial materials. OrmoComp lowered the viscosity by mixing with Ormothin (micro-resist technology GmbH, Germany) in a 3:1 ratio. The process conditions for both commercial materials were derived by applying the SPO, as shown in Table S3, confirming that both the developed and commercial resins could be successfully manufactured in the DLP system using SPO.

The scaffold was fabricated with the DLP system using three resins along with IP(8:2) as the development material. L-132 cells, which are normal lung cells, were cultured on a well plate with the three types of scaffolds. L-132 cells were seeded in 500 μL at a concentration of 5 × 10^4^ cells/cm^2^. After culturing for 24 h, the sample was treated with 800 μL dimethyl sulfoxide (Sigma-Aldrich). A treatment with 20 μL of MTT (Sigma-Aldrich) was performed for 4 h. The absorbance was measured at 540 nm using a microplate reader (Asys UVM 340; Biochrom Ltd., Cambridge, UK) to confirm cell viability^59,60^.

**Figure 9:**
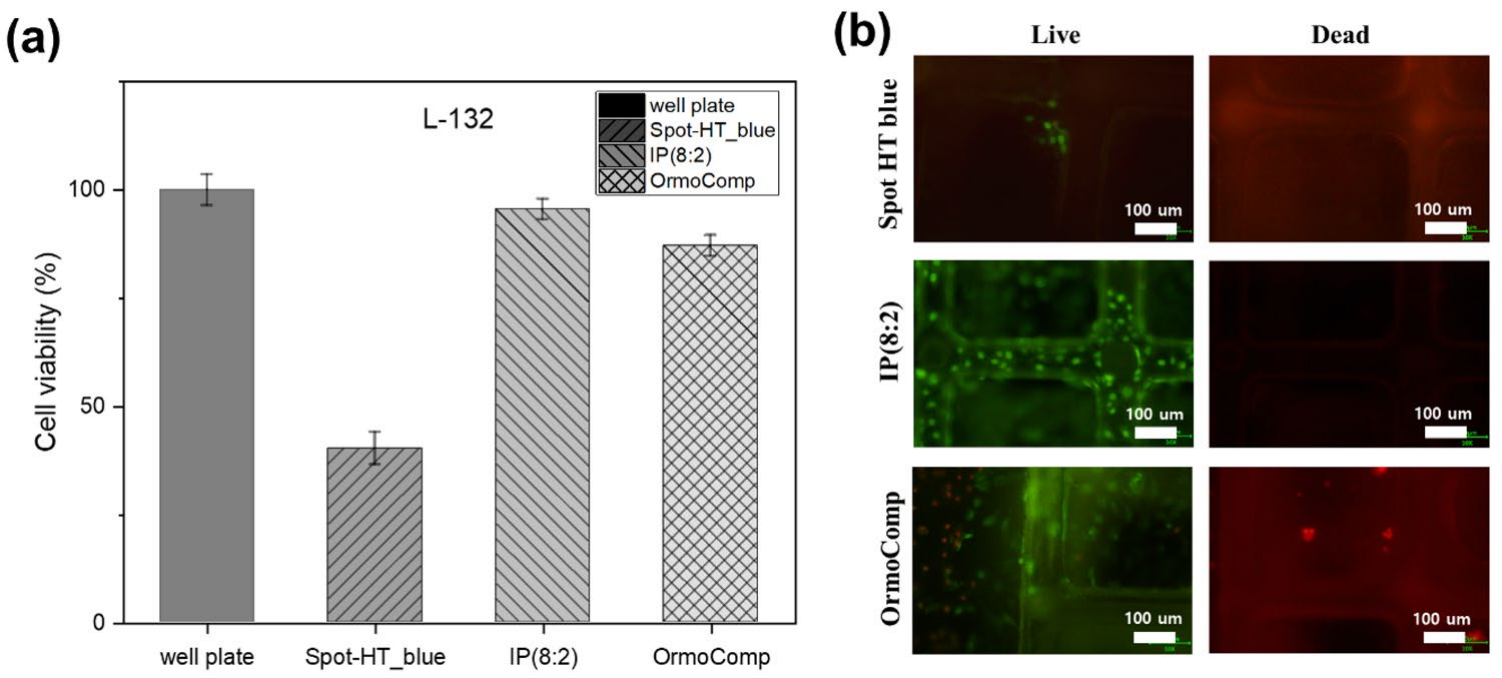
(**a**) L-132 cell viability results according to photopolymerization resin. (**b**) Cell observation results of the 3D scaffold of resins: Spot-HT blue, IP(8:2), OrmoComp.

A live/dead cell assay (BioVision, CA, USA) was used to observe cell adhesion and cytotoxicity in the developed materials. L-132 cells (5 × 10^4^ cells/mL) were cultured in Spot- HT blue, IP(8:2), and OrmoComp for 3 days. In sequence, cells were treated with Live-Dye, a cell-permeable green fluorescent dye, and propidium iodide, a non-permeable red fluorescent dye. The cells were observed using a Nikon Eclipse TS100led Trinocular fluorescence microscope (Nikon Corporation, Tokyo) with a band-pass filter (detect fluorescein and rhodamine), as shown in Fig. 9b.

The cell viability of the resin is shown in Fig. 9a. The cell culture images of Spot-HT, IP(8:2), and OrmoComp resin were produced using the DLP system. IP (8:2), an isosorbide-based resin, exhibited a high cell viability of 95.5%, confirming the biocompatibility of the developed resin. The values of OrmoComp and Spot-HT_blue were lower than those of IP(8:2). The environment for cell incubation of IP(8:2) was better than that of the other two structures. As shown in Fig. 9b, live and dead staining was performed to investigate the cell adhesion and cytotoxicity of the developed materials for 3D cell culture. The results indicated that the IP(8:2) materials increased cell adhesion compared to the Spot-HT blue and OrmoComp surfaces. In addition, living cells were observed in cells cultured in the pillars of the IP(8:2) scaffold. No dead cells were observed in the IP(8:2) scaffold. This suggests that IP(8:2) does not cause damage to cells, and the material is biocompatible. In contrast, the Spot-HT blue scaffold showed a few living cells, but almost no attached cells in pillars of the scaffold. Furthermore, the OrmoComp scaffold had dead cells. Our findings indicate that the developed IP(8:2) materials can be potentially used for 3D cell culture, regeneration, and drug screening in biomedical fields.

## Conclusion

This study derived process conditions using the SPO for application in the DLP system of an isosorbide-based biocompatible photocurable resin. The three typical problems in the DLP system are (1) initial adhesion, (2) poor resin recoating, and (3) over-cure and incomplete curing caused by inappropriate exposure conditions. Prevention of the identified problems is essential for the successful use of the DLP system. SPO is constructed to minimize trial and error by considering the representative problems in advance.

In the first sequence, a dataset of the photopolymerized resin thickness according to the exposure energy was obtained. In the second sequence, the resin behavior during the recoating process according to the resin viscosity and build plate movement was analyzed mathematically. Moreover, the availability of the resin and the required time were determined. The low-viscosity resin has high manufacturability and is advantageous for rapid manufacturing. In the developed IP(8:2) resin, 5.9 s were required for a one-layer cycle when manufacturing at a build plate speed of 100 mm/min in the DLP system used in the experiment. In the third sequence, the adhesion conditions of the first layer to the build plate were quantitatively evaluated using a direct method. At a processing speed of 100 mm/min for the IP(8:2) resin, the appropriate first layer exposure time was 2500 ms. Appropriate first-layer adhesion conditions prevented excessive adhesion and deformation problems.

In the last sequence, the appropriate level of overlap conditions required for additive manufacturing was confirmed. At a processing thickness of 100 µm, the over-cure required a minimum exposure time of 1400 ms to fabricate a 500-µm thick overhang structure. Three-step cleaning and UV curing were used as post-processing methods to prevent damage to the fabricated structure and increase durability.

A test artifact was generated to evaluate the derived process conditions. Furthermore, the biocompatibility of the developed resin was confirmed through cell experiments, and the applicability of the SPO to commercial resins was guaranteed.

Logical and efficient optimization of workflows in the manufacturing field reduces the possibility of process failure and waste of time and money. In this study, an SPO applied to an overall DLP system was proposed. The proposed method reduces process failure in the DLP system, as well as the waste of resources and time, thereby increasing its range of applications. In the future, it is necessary to add a system that heats high-viscosity resin so that it does not affect the manufacturing speed of the DLP system. The advanced SPO considering the heating system, is expected to increase the possibility of using high-viscosity resin in the DLP system.

## Supplementary Information

Supplementary Information.

## Data Availability

All data generated or analyzed during this study are included in this published article and its supplementary information files. The datasets used and/or analyzed during the current study are available from the corresponding author on reasonable request.

## References

[1] Ambrosi A, Pumera M (2016). 3D-printing technologies for electrochemical applications. Chem. Soc. Rev..

[2] Camposeo A, Persano L, Farsari M, Pisignano D (2019). Additive manufacturing: Applications and directions in photonics and optoelectronics. Adv. Opt. Mater..

[3] Moroni L (2018). Biofabrication: A guide to technology and terminology. Trends Biotechnol..

[4] Udofia EN, Zhou W (2020). 3D printed optics with a soft and stretchable optical material. Addit. Manuf..

[5] Gross B, Lockwood SY, Spence DM (2017). Recent advances in analytical chemistry by 3D printing. Anal. Chem..

[6] Gul JZ (2018). 3D printing for soft robotics—a review. Sci. Technol. Adv. Mater..

[7] Truby RL, Lewis JA (2016). Printing soft matter in three dimensions. Nature.

[8] Vaezi M, Seitz H, Yang S (2013). A review on 3D micro-additive manufacturing technologies. Int. J. Adv. Manuf. Technol..

[9] Choi JW (2021). A three-dimensional liquid-based exchangeable gradient osmosis chip for a permeability controllable microfluidic device. ACS Appl. Polym. Mater..

[10] Lu Y, Mapili G, Suhali G, Chen S, Roy K (2006). A digital micro-mirror device-based system for the microfabrication of complex, spatially patterned tissue engineering scaffolds. J. Biomed. Mater. Res. Part A:.

[11] Bagheri A, Jin J (2019). Photopolymerization in 3D printing. ACS Appl. Polymer Mater..

[12] You S, Wang P, Schimelman J, Hwang HH, Chen S (2019). High-fidelity 3D printing using flashing photopolymerization. Addit. Manuf..

[13] Warr C (2020). Biocompatible PEGDA resin for 3D printing. ACS Appl. Bio Mater..

[14] Waheed S (2016). 3D printed microfluidic devices: Enablers and barriers. Lab Chip.

[15] Bhattacharjee N, Urrios A, Kang S, Folch A (2016). The upcoming 3D-printing revolution in microfluidics. Lab Chip.

[16] Au AK, Huynh W, Horowitz LF, Folch A (2016). 3D-printed microfluidics. Angew. Chem. Int. Ed..

[17] He, Y., Wu, Y., Fu, J. z., Gao, Q. & Qiu, J. J. Developments of 3D printing microfluidics and applications in chemistry and biology: A review. *Electroanalysis* **28**, 1658–1678 (2016).

[18] Smith PT (2019). Additive manufacturing of bovine serum albumin-based hydrogels and bioplastics. Biomacromol.

[19] Santoliquido O, Colombo P, Ortona A (2019). Additive Manufacturing of ceramic components by Digital Light Processing: A comparison between the “bottom-up” and the “top-down” approaches. J. Eur. Ceram. Soc..

[20] Weems AC, Pérez-Madrigal MM, Arno MC, Dove AP (2020). 3D printing for the clinic: Examining contemporary polymeric biomaterials and their clinical utility. Biomacromol.

[21] Melchels FP, Feijen J, Grijpma DW (2010). A review on stereolithography and its applications in biomedical engineering. Biomaterials.

[22] Hu, K., Wei, Y., Lu, Z., Wan, L. & Li, P. Design of a shaping system for stereolithography with high solid loading ceramic suspensions. *3D Print. Addit. Manuf.* **5**, 311–318 (2018).

[23] Lian Q, Yang F, Xin H, Li D (2017). Oxygen-controlled bottom-up mask-projection stereolithography for ceramic 3D printing. Ceram. Int..

[24] Behroodi E, Latifi H, Najafi F (2019). A compact LED-based projection microstereolithography for producing 3D microstructures. Sci. Rep..

[25] Wang, Z., Martin, N., Hini, D., Mills, B. & Kim, K. Rapid fabrication of multilayer microfluidic devices using the liquid crystal display-based stereolithography 3D printing system. *3D Print. Addit. Manuf.* **4**, 156–164 (2017).

[26] Ahn D, Stevens LM, Zhou K, Page ZA (2020). Rapid high-resolution visible light 3D printing. ACS Cent. Sci..

[27] Lee JH, Prud'Homme RK, Aksay IA (2001). Cure depth in photopolymerization: Experiments and theory. J. Mater. Res..

[28] Zheng X (2012). Design and optimization of a light-emitting diode projection micro-stereolithography three-dimensional manufacturing system. Rev. Sci. Instrum..

[29] Choong YYC, Maleksaeedi S, Eng H, Su P-C, Wei J (2017). Curing characteristics of shape memory polymers in 3D projection and laser stereolithography. Virtual Phys. Prototyping.

[30] Aduba DC (2019). Vat photopolymerization 3D printing of acid-cleavable PEG-methacrylate networks for biomaterial applications. Mater. Today Commun..

[31] Guerra AJ (2019). Optimization of photocrosslinkable resin components and 3D printing process parameters. Acta Biomater..

[32] Choi, J. W. *et al.* Optimization of the projection microstereolithography process for a photocurable biomass-based resin. *3D Print. Addit. Manuf.* **8**, 293–301 (2021). 10.1089/3dp.2020.0173 PMC9828617 36654934

[33] Kim S, Cho JK, Shin S, Kim B-J (2015). Photo-curing behaviors of bio-based isosorbide dimethacrylate by irradiation of light-emitting diodes and the physical properties of its photo-cured materials. J. Appl. Polym. Sci..

[34] Wu J (2011). Isohexide derivatives from renewable resources as chiral building blocks. Chemsuschem.

[35] Rose M, Palkovits R (2012). Isosorbide as a renewable platform chemical for versatile applications—quo vadis?. Chemsuschem.

[36] Fenouillot F, Rousseau A, Colomines G, Saint-Loup R, Pascault J-P (2010). Polymers from renewable 1, 4: 3, 6-dianhydrohexitols (isosorbide, isomannide and isoidide): A review. Prog. Polym. Sci..

[37] Han D-H, Kim M-J, Jun E-J, Kim J-B (2012). Salivary bisphenol-A levels due to dental sealant/resin: a case-control study in Korean children. J. Korean Med. Sci..

[38] Du Y, Zhang J, Zhou C (2016). Synthesis and properties of waterborne polyurethane-based PTMG and PDMS as soft segment. Polym. Bull..

[39] Deng Y, Zhou C, Zhang Q, Zhang M, Zhang H (2020). Structure and performance of waterborne polyurethane-acrylate composite emulsions for industrial coatings: Effect of preparation methods. Colloid Polym. Sci..

[40] Park J (2014). Conformal phase masks made of polyurethane acrylate with optimized elastic modulus for 3D nanopatterning. J. Mater. Chem. C.

[41] Choi JW, Wicker RB, Cho SH, Ha CS, Lee SH (2009). Cure depth control for complex 3D microstructure fabrication in dynamic mask projection microstereolithography. Rapid Prototyping J..

[42] Urrios A (2016). 3D-printing of transparent bio-microfluidic devices in PEG-DA. Lab. Chip.

[43] Ian Gibson, I. G. *Additive manufacturing technologies 3D printing, rapid prototyping, and direct digital manufacturing*. 498 (Springer, 2015).

[44] Emami MM, Barazandeh F, Yaghmaie F (2014). Scanning-projection based stereolithography: Method and structure. Sens. Actuators, A.

[45] Jacobs, P. F. *Rapid prototyping & manufacturing: fundamentals of stereolithography*. (Society of Manufacturing Engineers, 1992).

[46] Caprioli M (2021). 3D-printed self-healing hydrogels via digital light processing. Nat. Commun..

[47] Luo, Y., Le Fer, G. l., Dean, D. & Becker, M. L. 3D printing of poly (propylene fumarate) oligomers: Evaluation of resin viscosity, printing characteristics and mechanical properties. *Biomacromolecules* **20**, 1699–1708 (2019). 10.1021/acs.biomac.9b00076 30807696

[48] Pang D, Cong H, Li X, Li H, Gao X (2021). Liquid-bridge flow between two slender plates: Formation and fluid mechanics. Chem. Eng. Res. Des..

[49] Men Y, Zhang X, Wang W (2009). Capillary liquid bridges in atomic force microscopy: Formation, rupture, and hysteresis. J. Chem. Phys..

[50] Ni Q, Crane N (2018). Controlling normal stiffness in droplet-based linear bearings. Micromachines.

[51] Tadrist L, Motte L, Rahli O, Tadrist L (2019). Characterization of interface properties of fluids by evaporation of a capillary bridge. R. Soc. Open Sci..

[52] Bowen J, Cheneler D (2020). Closed-form expressions for contact angle hysteresis: capillary bridges between parallel platens. Colloids Interfaces.

[53] Nguyen HNG, Zhao C-F, Millet O, Selvadurai A (2021). Effects of surface roughness on liquid bridge capillarity and droplet wetting. Powder Technol..

[54] Brulin, S., Roisman, I. V. & Tropea, C. Fingering instability of a viscous liquid bridge stretched by an accelerating substrate. *J. Fluid Mech.* **899** (2020).

[55] Li X, Mao H, Pan Y, Chen Y (2019). Mask video projection-based stereolithography with continuous resin flow. J. Manuf. Sci. Eng..

[56] Noel AC, Guo H-Y, Mandica M, Hu DL (2017). Frogs use a viscoelastic tongue and non-Newtonian saliva to catch prey. J. R. Soc. Interface.

[57] Ward T (2011). Capillary-pressure driven adhesion of rigid-planar surfaces. J. Colloid Interface Sci..

[58] Gong H, Beauchamp M, Perry S, Woolley AT, Nordin GP (2015). Optical approach to resin formulation for 3D printed microfluidics. RSC Adv..

[59] Kim G-J, Lee K-J, Choi J-W, An JH (2021). Drug evaluation based on a multi-channel cell chip with a horizontal co-culture. Int. J. Mol. Sci..

[60] Nunewar SN (2020). Synthesis of 1-(Indol-2-yl)-phenoxazine hybrids from quinacetophenone precursors and their biological evaluation as DNA intercalating agents. J. Mol. Struct..

