# Supplementary material for: Sequential process optimization for a digital light processing system to minimize trial and error
Source: Sci Rep. 2022 Aug 8;12:13553. doi: 10.1038/s41598-022-17841-5 (PMC9360010; doi:10.1038/s41598-022-17841-5)
Supplement: Supplementary file 1 — Supplementary Information. [file 41598_2022_17841_MOESM1_ESM.pdf]

Sequential process optimization for a digital light processing system to minimize trial and error

*Jae Won Choi[1,2], Gyeong-Ji Kim[3], Sukjoon Hong[2], Jeung Hee An[3], Baek-Jin Kim[4,5], Cheol Woo Ha[1]**

[1] Advanced Joining and Additive Manufacturing R&D Department, Korea Institute of Industrial Technology, 113-58, Seohaean-ro, Siheung-si, 15014, Republic of Korea

[2] Department of Mechanical Engineering, BK21 FOUR ERICA-ACE center, Hanyang University, 55 Hanyangdaehak-ro, Ansan, 15588, Republic of Korea,

[3] Department of Food and Nutrition, KC University, 47, 24-Gil, Kkachisan–Ro, Seoul, 07661, Republic of Korea

[4] Green Chemistry and Materials Group, Korea Institute of Industrial Technology, 35-3, Seobuk-gu, Cheonan, 31056, Republic of Korea.

[5] Department of Green Process and System Engineering, Korea University of Science and Technology (UST), 35-3, Seobuk-gu, Cheonan, 31056, Republic of Korea.

*Co-corresponding Author

Cheol Woo Ha

Tel : +82-31-8084-8827

E-mail address : cwha@kitech.re.kr

Supporting information

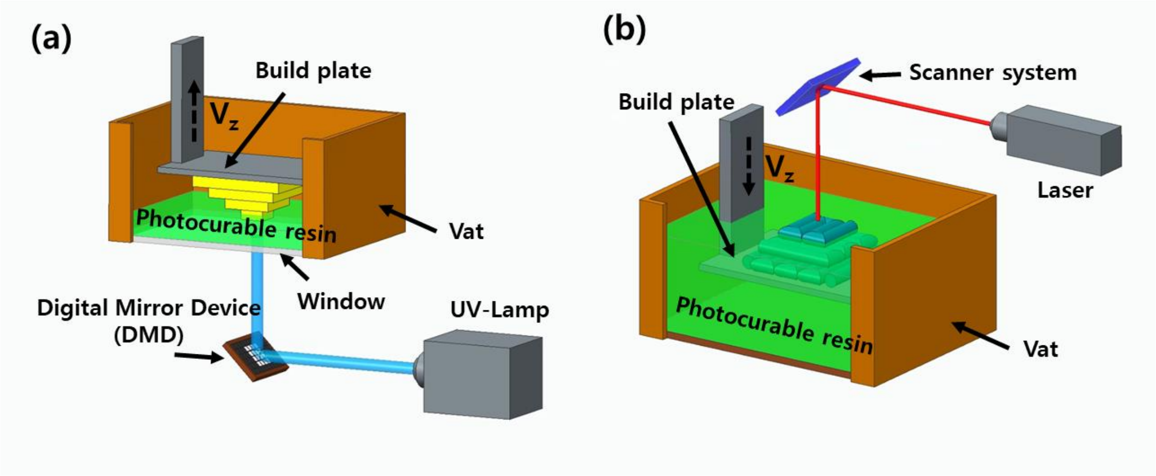

Figure S1. Schematic diagram of VPP. (a) Bottom-up method, (b) top-down method.

The VPP method is divided into bottom-up and top-down approaches according to the build plate and vat configuration. As shown in Figure S1 (b), the top-down approach is suitable for manufacturing large parts. However, a large amount of resin is required and cleaning is challenging. In addition, the fabrication of closed parts is challenging and controlling the curing thickness is difficult because it is sensitive to the recoating system.

The bottom-up approach can be utilized with a small amount of resin and effectively prevents material waste. Because the gap between the vat and the build plate is controlled, the cured thickness of each layer is maintained constant. Therefore, high-precision products are fabricated in the z-direction. However, a large separation force is generated when the build plate for the next layer is raised because a negative pressure is generated during the layer-curing process in this approach. Separation forces cause product defects. In addition, in the bottom-up approach, the photopolymerization reaction occurs from the bottom of the resin. Therefore, the ratio of the medium in the resin is limited and the slurry does not accumulate. Therefore, the selection of material and viscosity is important for the bottom-up approach.

Table S1. Comparison of top-down and bottom-up approaches.

	Top-down	Bottom-up
**Layer thickness control**	Difficult	Easy
**Recoating mechanism**	Necessary	Unnecessary
**Required resin**	Large	Small
**Cleaning**	Difficult	Easy

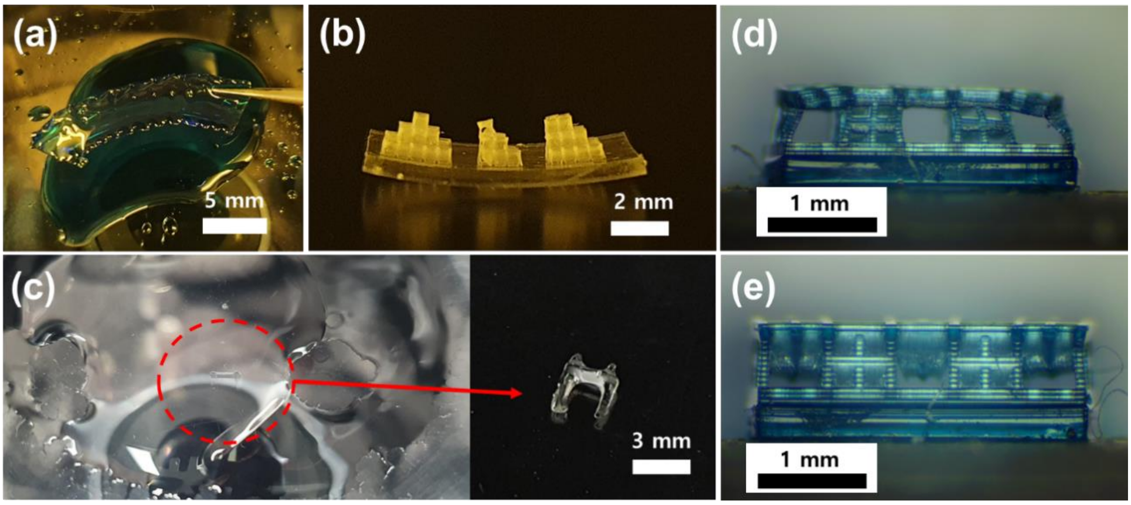

Figure S2. Fabrication error types: (a) layer slip, (b) deformation, (c) recoating and first layer adhesion errors, (d) insufficient polymerization, (e) excessive polymerization.

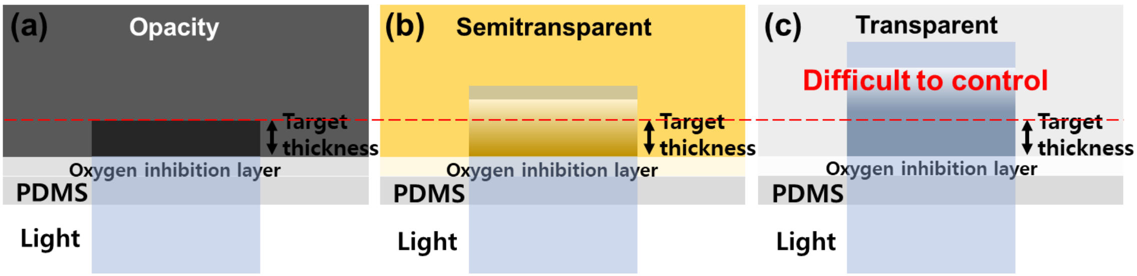

Figure S3. Photopolymerization control according to the transparency of the resin: (a) opaque resin, (b) semitransparent resin, (c) transparent resin.

In some cases, bio-devices must be transparent for internal observation. In the DLP system, as shown in Figure S3(a), opaque materials block light transmission, making it easier to control the amount of light polymerization targeted. However, a more transparent resin transmits more light, as shown in Figure S3(c), hindering the control of the curing thickness. Thus, creating microscopic structures with transparent resin is challenging.

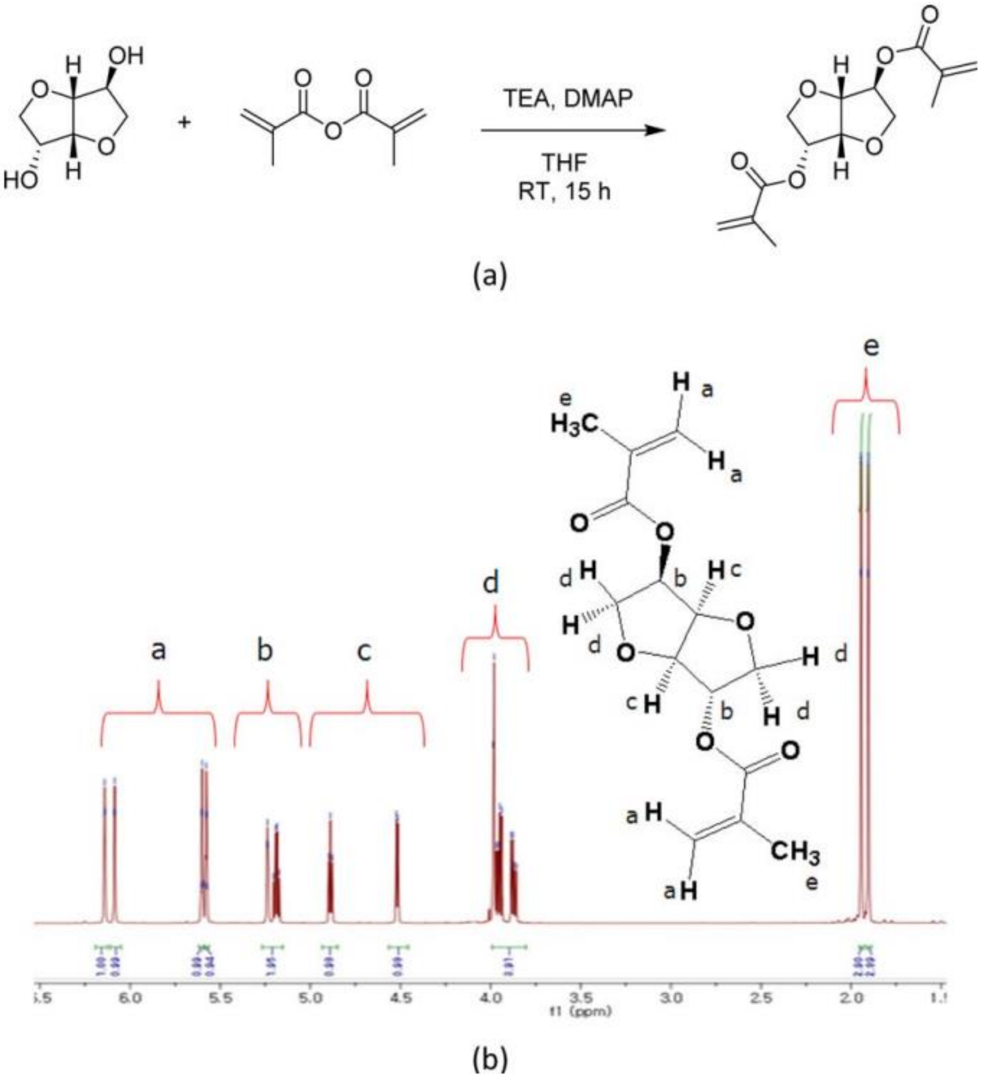

Figure S4. (a) Reaction scheme for ISDM from isosorbide and (b) proton nuclear magnetic resonance (^{1}H NMR) spectra of the synthesized ISDM.

Isosorbide (2.92 g, 20 mmol), TEA (44 mmol, 2.2 equivalents), and DMAP (4.4 mmol, 0.22 equivalents) were placed in a round-bottomed flask, and dry THF (60 mL) was added. Methacrylic anhydride (44 mmol, 2.2 equivalents) in dry THF (30 mL) was added dropwise. The reaction mixture was stirred under an N_2 atmosphere at room temperature (25 °C) for 15 h (Figure 2(a)). THF was evaporated under reduced pressure after the completion of the reaction. Synthetic ISDM was characterized by FT-NMR (Avance III, Bruker, Germany) and gas chromatography–mass spectrometry (GC-MS) (5975, Agilent, USA). Chemical shifts (d) and coupling constants (J) are expressed in parts per million (ppm) and hertz (Hz), respectively.

The mass spectrometer was operated in ESI mode (m/z<2,000). ^{1}H-NMR (400 MHz, CDCl3): d 6.14 (s, 1H, He), 6.09 (s, 1H, He), 5.60 (s, 1H, He), 5.58 (s, 1H, He), 5.24 (br, 1H, Hb), 5.19 (q, J 5 4 Hz, 1H, Hb), 4.89 (t, J 5 4.0 Hz, 1H, Hc), 4.52 (d, J 5 4.0 Hz, 1H, Hc), 3.98 (br, 2H, Hd), 3.95 (dd, J 5 8.0, 4.0 Hz, 1H, Hd), 3.87 (dd, J 5 8.0, 4.0 Hz, 1H, Hd), 1.94 (s, 3H, He), 1.90 (s, 3H, He) (Figure 2(b)). 13C-NMR (100 MHz, CDCl3): d 166.9, 166.6, 135.9, 135.8, 126.7, 126.5, 86.2, 82.1, 78.4, 74.4, 73.6, 70.8, 18.5, 18.4. The mass spectrum (ESI) m/z calculated for C14H19O6 [M 1 H]1 283.1 was 283.1.

Table S2. Blend ratios (w/w) for ISDM and PTMG PUA.

IP resin	Weight ratio (w/w)
1. ISDM : PUA	0 : 10
2. ISDM : PUA	2 : 8
3. ISDM : PUA	8 : 2
4. ISDM : PUA	10 : 0

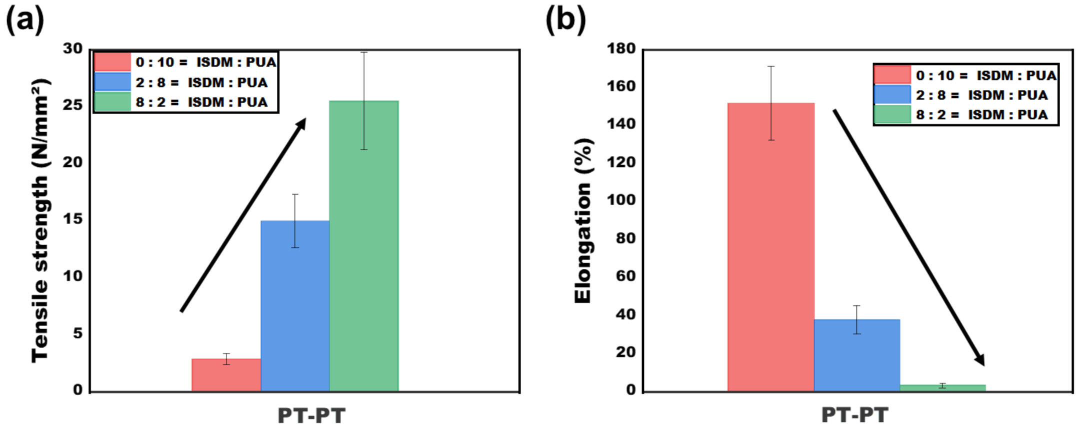

Figure S5. (a) Tensile strength result for blend of ISDM and PTMG PUA, (b) Elongation result for blend of ISDM and PTMG PUA.

A universal test machine (UTM) was used to measure the tensile strength and elongation at break of curable films for the blends of ISDM and PTMG PUA. Specimen films for UTM measurement were prepared with weight fractions of ISDM and PTMG PUA, as shown in Table S2 (1-3). After filling a 500-μm thick frame mold made of Teflon material with UV curable resin, all blends were cured by an irradiator with a UV-infrared double zone. The UV wavelengths and intensities used at this time were as follows: UVA (230–390 nm): 573.608 mW/cm^2, UVB (280–320 nm): 267.316 mW/cm^2, UVC (250–260 nm) conditions: 42.510

mW/cm. After UV irradiation, all specimens were removed from the mold, and the tensile strength and elongation were measured. A 100-N load cell was used, and the crosshead was measured at a speed of 12.5 mm/min to obtain the stress-strain curve. All blends were confirmed to be transparent and fully cured. As shown in Figure S5, the higher the ISDM content, the higher the tensile strength, and the higher the PTMG PUA content, the higher the elongation. In particular, when the ISDM content reached 20% (ISDM : PTMG PUA=2:8), the elongation increased by 35% and the tensile strength decreased to approximately 50%, indicating a trade-off relationship.

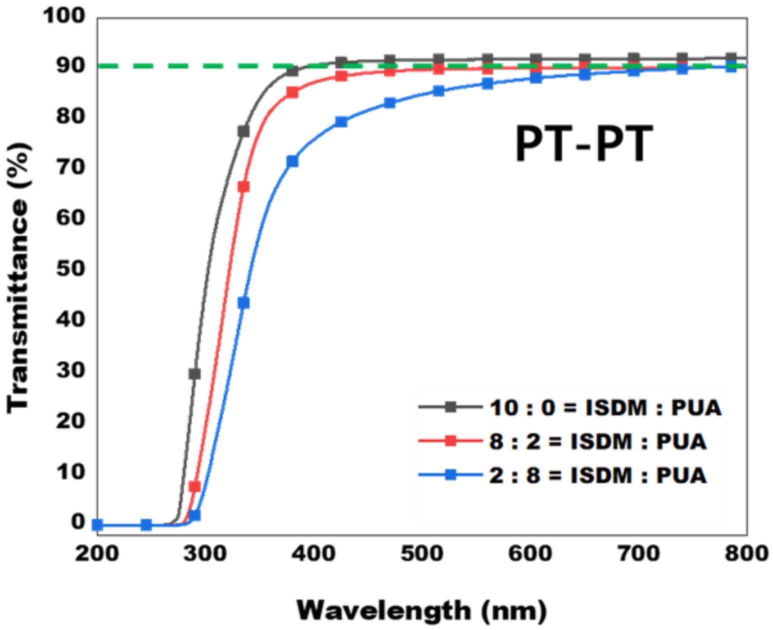

Figure S6. Transmittance results for blends of ISDM and PTMG PUA.

UV-visible spectra were measured using a UV-1800 UV-VIS spectrometer (SIMADZU) to confirm the difference in light transmittance of the blend materials. The transmittance was measured in the range of 200–800 nm. The specimen film for the transmittance measurement was prepared as shown in Table S2 (2-4). The thickness of the film specimen was 500 μm, which was very thick. This was because the film thickness did not show a significant difference, as it had a high transmittance value of 92% or more when it was 100 μm or less. As a result of measuring the light transmittance of all specimens, ISDM showed a high transmittance of 90% or more in the wavelength range of 400 to 800 nm. In addition, as the PTMG PUA content increased, a transmittance of 75–80% was measured in the 400–600 nm wavelength range. The specimens of all blend materials were prepared in the same manner as the tensile strength specimens and cured under the same conditions.

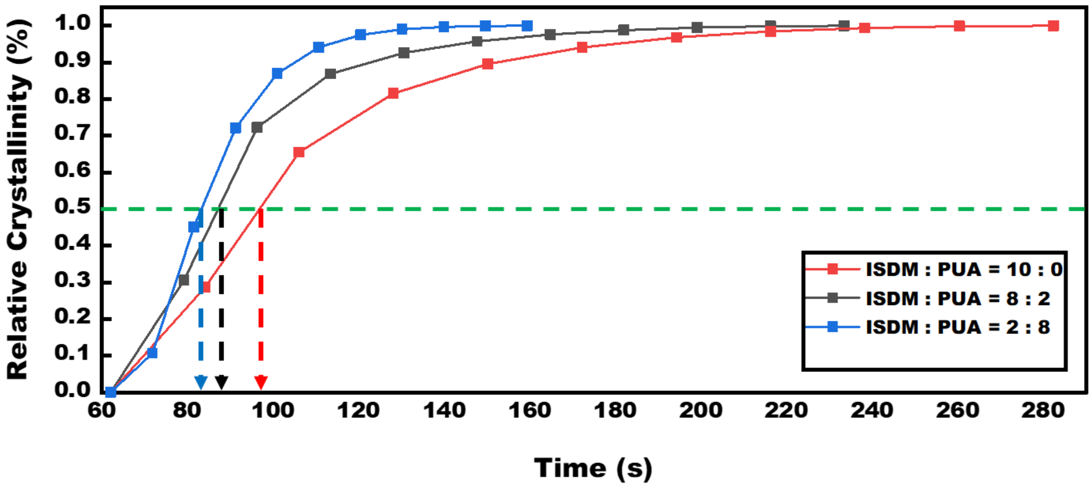

Figure S7. Photo-curing conversion of PTMG PUA and ISDM.

The photo-polymerization reaction was measured isothermally using PerkinElmer's differential photo-calorimeter DSC 8000 and OmniCure Series 2000 irradiating a luminous intensity of 21.4 mJ/cm^2. The amount of light in the OmniCure Series 2000 was measured using the R2000 radiometer of OmniCure. A sample of 10 mg of each blend material was filled into a shallow open aluminum pan, covered with UV-passed quartz, and measured under a nitrogen atmosphere. A Level 2 optical path of 21.4 mJ/cm2 was selected and measured.

The photo-curing rate according to the content of each PUA sample is shown in Figure S7. The photo-curing rate tended to increase with the addition of ISDM owing to the high viscosity of ISDM. The t1/2, which is the time required for half-curing conversion, is a key index that determines the degree of curing rate for all blends of UV-curable materials. This can be calculated using the thermal energy generated during curing. That is, when the amount of heat generated in the entire curing process is taken as the denominator, the value obtained by taking the amount of heat generated until half-curing time is the numerator. In our blend materials, the rank of all blend materials participating in the photo-curing process was ISDM : PUA=10:0 > 8:2 > 2:8 for t1/2 values of 95 s, 87 s, and 82 s, respectively. According to the t1/2 results, the curing was confirmed to proceed more slowly as the content of ISDM increased.

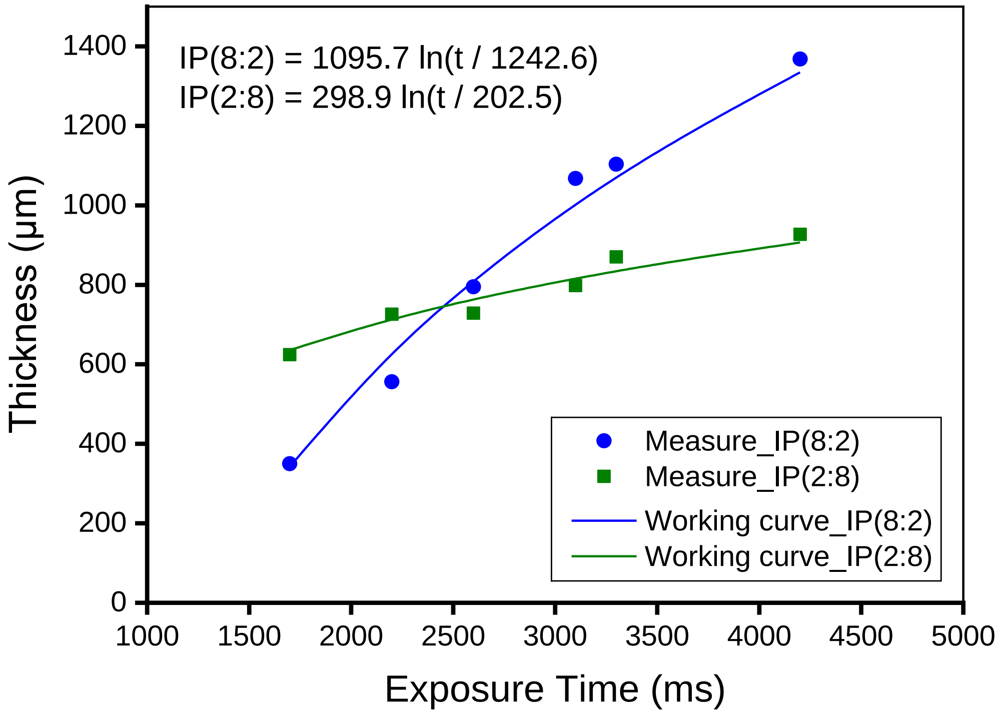

Figure S8. Comparison of measured and theoretical thicknesses based on working curve equation.

A single layer was fabricated under each exposure time condition randomly selected (1,700, 2,200, 2,600, 3,100, 3,300, and 4,200 ms), and the cured thickness was measured.

Figure S8 shows a comparison of the theoretical and measured values, where the measured values of both IP(2:8) and IP(8:2) are located in the error area from the theoretical values. Consequently, reasonable photopolymerization properties of the resins were confirmed by the proposed method. The curing thicknesses according to the exposure time of both the IP(2:8) and IP(8:2) resins obtained from the working curve equation can be used in future sequences.

Table S3. Optimal process conditions were obtained using SPO for IP (8:2) resin and two commercial resins (Spot-HT blue, OrmoComp).

	IP(8:2)	Spot-HT blue	OrmoComp
Layer thickness (μm)	100	100	100
Z-lift speed (mm/min)	100	100	50
Initial exposure time (ms)	2,500	1,600	2,200
Exposure time (ms)	1,400	500	900
